# Amino Acid Profiles in Dried Blood Spots and Clinical Outcomes in Selective Fetal Growth Restriction: A Twin-Pair Study

**DOI:** 10.3390/ijms27209156

**Published:** 2026-10-15

**Authors:** Vitaliy V. Chagovets, Alisa O. Tokareva, Natalia A. Frankevich, Anastasia V. Novoselova, Marina N. Yushina, Fadi Hajjar, Chupalav M. Eldarov, Olga V. Michailova, Tamara E. Karapetyan, Irina I. Ryumina, Vladimir E. Frankevich, Gennady T. Sukhikh

**Affiliations:** 1 National Medical Research Center for Obstetrics Gynecology and Perinatology Named After Academician V.I. Kulakov of the Ministry of Healthcare of Russian Federation, 117997 Moscow, Russia; 2 Department of Obstetrics, Gynecology, Perinatology and Reproductology, Institute of Professional Education, Federal State Autonomous Educational Institution of Higher Education I.M. Sechenov First Moscow State Medical University of the Ministry of Health of the Russian Federation, 119991 Moscow, Russia

**Keywords:** selective fetal growth restriction, dried blood spots, mass spectrometry, monochorionic twins, dichorionic twins, amino acids, neonatal outcomes, over-representation analysis

## Abstract

Selective fetal growth restriction (sFGR) remains a leading cause of perinatal morbidity in multiple pregnancies; however, its pathogenesis is not yet fully elucidated. Investigating amino acid metabolism in weight-discordant twins may uncover critical metabolic pathways involved in this condition, given the essential roles of amino acids in fetal growth and energy supply. The use of dried blood spots (DBSs) enables the minimally invasive, retrospective evaluation of neonatal metabolic profiles, providing a unique opportunity to identify sFGR-associated signature and clarify differences between monochorionic (MC) and dichorionic (DC) placentation. In the present study, we profiled 23 amino acids in DBS from 23 twin pairs (11 discordant and 12 non-discordant, defined by postnatal birth-weight discordance), stratified by chorionicity, to identify specific metabolic signatures associated with sFGR and to evaluate their temporal dynamics and clinical significance. Our findings suggest that chorionicity is associated with distinct amino acid signatures of postnatal birth-weight discordance. In MC twins, the larger co-twin displayed significantly elevated glycine, α- and γ-aminobutyric acids, serine, asparagine, and citrulline, alongside reduced proline and tryptophan. In DC twins, the larger co-twin exhibited higher α-aminobutyric acid, valine, leucine, arginine, and citrulline, whereas glycine was elevated in the smaller co-twin. Pathway enrichment analysis further suggested the enrichment of amino acid transport and protein synthesis pathways in MC postnatal discordance and nitric oxide (NO)-dependent signaling in DC postnatal discordance. Longitudinal follow-up showed a statistically significant attenuation of intrapair differences for several amino acids by the second time point. All 11 discordant pairs were retrospectively confirmed to fulfil the Delphi criteria for sFGR. These findings are exploratory and hypothesis-generating.

## 1. Introduction

Selective fetal growth restriction (sFGR) occurs in 10–26% of monochorionic [1] and 5–11% of dichorionic twin pregnancies [2,3] and remains a leading cause of perinatal morbidity and mortality. According to the current understanding, the pathogenesis of sFGR differs fundamentally depending on chorionicity: in monochorionic twins, the key mechanism is uneven distribution of blood flow through vascular anastomoses of the shared placenta, whereas in dichorionic twins the underlying mechanisms are similar to those in singleton pregnancies and are driven by placental insufficiency of each fetus’s own placenta [4,5,6].

In recent years, the international medical community has reached a consensus on the unification of terminology for growth restriction of one fetus in multiple pregnancies. The pivotal document defining contemporary diagnostic criteria is the 2019 Delphi consensus led by A. Khalil and colleagues [7]. Within this consensus, a group of international experts developed a definition of selective fetal growth restriction (sFGR) that applies regardless of chorionicity. Separate diagnostic criteria were established for monochorionic and dichorionic twins, reflecting differences in their pathophysiology: for dichorionic twins, a diagnosis of sFGR requires at least two of three additional criteria, whereas for monochorionic twins at least two of four criteria are needed. It is important to note that the Delphi consensus represents a consensus-based research framework rather than a fully validated diagnostic standard; the original publication explicitly indicated the need for subsequent validation. Furthermore, the Delphi criteria differ from conventional clinical definitions of sFGR, which often rely on broader parameters such as EFW < 10th percentile, AC < 10th percentile, inter-twin discordance ≥20–25%, or abnormal umbilical artery Doppler, without the specific combination rules of the Delphi procedure. This approach was reinforced in the updated practical guidelines of the International Society of Ultrasound in Obstetrics and Gynecology (ISUOG Practice Guidelines) [8], which apply the term sFGR to both monochorionic and dichorionic twins complicated by growth restriction of one fetus. Recent publications, including those in the leading journal *Ultrasound in Obstetrics & Gynecology*, also actively use the term sFGR in the context of dichorionic twins [9].

Despite considerable advances in prenatal diagnosis and monitoring [10,11] , the molecular mechanisms that determine why one twin grows less than the other under identical genetic and maternal conditions remain incompletely understood. Twin pairs, especially monochorionic, genetically identical twins, provide a natural “controlled” model in which inter-twin differences can be attributed primarily to placental dysfunction rather than to genetic background or maternal nutrition. One promising approach for assessing the metabolic status of newborns is the analysis of dried blood spots (DBSs). This method is widely employed in neonatal screening programs for inherited metabolic disorders. Since 2023, the Russian Federation has implemented expanded neonatal screening, which uses tandem mass spectrometry to determine markers of 29 inherited diseases in DBSs [12]; as of 2024, the program has been extended to cover more than 40 conditions. For preterm and low-birth-weight infants, who constitute the majority of patients with sFGR, the use of DBSs is particularly valuable: the analysis requires only 10–20 µL of capillary blood, thereby avoiding painful venipunctures, reducing the risk of iatrogenic anemia, and minimizing stress for the infant. In recent years, tandem mass spectrometry of DBSs has become the standard for the quantitative determination of amino acids, acylcarnitines, and other metabolites, enabling metabolomic screening without additional invasive procedures.

International studies indicate that characteristic metabolic alterations are observed in fetal growth restriction (FGR). A systematic review published in 2024, which included 48 studies and 825 non-duplicated metabolites, identified three amino acid metabolism pathways and one lipid metabolism pathway significantly associated with FGR/SGA, including phenylalanine metabolism in newborn dried blood spots [13]. In a prospective longitudinal study, Huang et al. 2023 [14] showed that a combination of L-phenylalanine, L-leucine, and L-isoleucine in the second trimester is a highly informative predictor of sFGR development (AUC reaching 0.878), while a combination of L-serine, L-histidine, and L-arginine in the first trimester correlates with long-term neurocognitive development [14]. The same authors also reported pronounced amino acid alterations in amniotic fluid in sFGR [15]. In this study, we use the term “postnatal birth-weight discordance” to describe the inter-twin difference in actual birth weights, calculated as Δm = 2 × (m_larger − m_smaller)/(m_larger + m_smaller) × 100. This measure was used to define the study groups. It is distinct from “prenatal EFW discordance”, which is one of the Delphi diagnostic criteria for sFGR and was calculated from ultrasound data. All discordant pairs in this study were retrospectively confirmed to fulfil the Delphi criteria for sFGR.

Data on birth-weight-discordant twins remain fragmentary and, most importantly, largely fail to account for chorionicity. Such studies are practically nonexistent in the Russian literature. Furthermore, it remains unclear whether alterations in the amino acid profile correlate with functional outcomes (timing of transition to enteral feeding, length of hospitalization, discharge weight) and whether they could serve as predictors of adverse outcomes. As noted above, DBS analysis is already integrated into routine neonatal practice in Russia, with samples collected from all newborns within the first 24–48 h of life. This creates a unique opportunity for retrospective and prospective analysis of metabolic profiles without additional blood sampling. The present study was designed to fill the aforementioned gaps. It is the first to conduct a comparative analysis of the DBS amino acid profile in twin pairs with and without sFGR, stratified by chorionicity (monochorionic vs. dichorionic) and to evaluate the association of metabolic disturbances with detailed clinical parameters, including functional care outcomes. Specifically, this study aimed to compare the amino acid profiles of capillary dried blood spots in twins from postnatally discordant and non-discordant pairs, stratified by chorionicity; to assess clinical differences between twins and their correlations with metabolic data; and to explore the potential practical significance of DBS amino acid analysis for future neonatal management strategies.

## 2. Results

### 2.1. Clinical Characteristics

#### 2.1.1. Postnatally Discordant Pairs

In postnatally discordant pairs (*n* = 11), the smaller twin had significantly lower birth weight (median 1160 g vs. 1840 g for the larger twin, *p* < 0.001), birth length (38 cm vs. 42 cm, *p* < 0.001), head circumference (28 cm vs. 31 cm, *p* = 0.004), and birth weight percentile (5th vs. 60th, *p* < 0.001). Discharge weight also differed significantly (1935 g vs. 2510 g, *p* < 0.001) (Table 1). All 11 postnatally discordant pairs were retrospectively confirmed to fulfil the Delphi criteria for sFGR (Supplementary Table S1). Among the 11 smaller twins from postnatally discordant pairs, 7 (64%) were SGA at birth (<10th percentile) and 5 (45%) had severe SGA (<3rd percentile). In contrast, none of the 12 smaller twins from non-discordant pairs were SGA. Thus, prenatal sFGR and postnatal SGA were not fully concordant: 4 of the 11 smaller twins from postnatally discordant pairs were not SGA at birth.

The postnatal birth-weight discordance between twins in the discordant group was −40% versus −9.4% in the non-discordant group (*p* < 0.001), the birth length difference was −10% versus −2.3% (*p* < 0.001), the head circumference difference was −7.1% versus −3% (*p* = 0.004), and the birth weight percentile difference was −156% versus −52% (*p* < 0.001). The difference in the initiation of nipple feeding was +30% versus 0% (*p* = 0.04), the difference in time to full nipple feeding was +22% versus 0% (*p* = 0.007), and the discharge weight difference was −26% versus −9.8% (*p* < 0.001) (Table 2).

No significant differences were found between monochorionic and dichorionic postnatally discordant pairs for the listed clinical parameters (all *p* > 0.05) (Table 3).

#### 2.1.2. Maternal and Placental Parameters

In mothers of postnatally discordant pairs, gestational age at delivery was significantly lower (32 weeks vs. 34 weeks, *p* = 0.01) and placental weight was significantly lower (544 g vs. 625 g, *p* = 0.02). In the subgroup of dichorionic postnatally discordant pairs, maternal and paternal ages were higher (39 years vs. 30 years, *p* = 0.01; paternal age 40 years vs. 35 years, *p* = 0.02). The frequency of gestational diabetes mellitus (GDM) in the discordant group was 45% versus 8% in the non-discordant group (*p* = 0.12), and the frequency of preeclampsia was 18% versus 25% (*p* = 0.94) (Table 4 and Table 5).

### 2.2. Amino Acid Profile

#### 2.2.1. Differences Between the Larger and Smaller Twins

The differences in amino acid concentrations between the larger and smaller twins were most pronounced in the first DBS sample collected on days 1–2 of life, before the establishment of full enteral feeding. This indicates that the observed metabolic differences are primarily of intrauterine origin and are not attributable to postnatal nutrition. In monochorionic postnatally discordant pairs (*n* = 6), the OPLS model yielded R^2^X = 0.57, R^2^Y = 0.85, and Q^2^Y = 0.59, with *p*(R^2^Y) = 0.06 and *p*(Q^2^Y) = 0.03 according to permutation tests. The larger twin exhibited significantly higher concentrations of glycine (FC = 1.18, FDR = 0.02), AABA (FC = 1.43, FDR = 0.006), GABA (FC = 1.51, FDR = 0.01), and serine (FC = 1.31, FDR = 0.048); levels of proline (FC = 0.80, FDR = 0.001) and tryptophan (FC = 0.81, FDR = 0.007) were lower. The differences in β-alanine (FC = 1.39, FDR = 0.22), asparagine (FC = 1.29, FDR = 0.16) and citrulline (FC = 1.16, FDR = 0.20) did not reach statistical significance (Figure 1, Table 6). The points were statistically significantly discriminated according to Hotelling’s test, with *p*-value = 0.02, large effect size d = 3.14 and calculated minimum sample size in each group *n* = 4 for power = 0.80.

In dichorionic postnatally discordant pairs (*n* = 5), the OPLS model yielded R^2^X = 0.42, R^2^Y = 0.96, and Q^2^Y = 0.86, with *p*(R^2^Y) = 0.03 and *p*(Q^2^Y) = 0.02 according to permutation tests. The larger twin showed increased levels of AABA (FC = 1.84, FDR = 0.01), valine (FC = 1.24, FDR = 0.04), leucine (FC = 1.23, FDR = 0.04) and citrulline (FC = 1.20, FDR = 0.04); arginine (FC = 1.67, FDR = 0.11) and serine (FC = 1.19, FDR = 0.17) were not significantly different. Glycine was higher in the smaller twin (FC = 0.87, FDR = 0.04) (Figure 2, Table 7). The points statistically were significantly discriminated according to Hotelling’s test, with *p*-value = 0.02, large effect size d = 2.59 and calculated minimum sample size in each group *n* = 5 for power = 0.80.

The combined OPLS model for all postnatally discordant pairs yielded R^2^X = 0.41, R^2^Y = 0.80, and Q^2^Y = 0.28, with *p*(R^2^Y) = 0.04, and *p*(Q^2^Y) = 0.04. Seven amino acids had VIP > 1: AABA, serine, asparagine, valine, leucine, citrulline, and tryptophan. AABA (FC = 1.51, FDR < 0.001), serine (FC = 1.15, FDR = 0.009) and citrulline (FC = 1.18, FDR = 0.04) were significantly higher in the larger twin; leucine (FC = 1.01, FDR = 0.50), asparagine (FC = 1.23, FDR = 0.50), and valine (FC = 1.12, FDR = 0.50) were also higher but not significantly; tryptophan (FC = 0.73, *p* = 0.19) was not statistically significantly lower (Figure 3, Table 8). Ellipses of points have a large discrimination with *p* = 0.05 for Hotelling’s test, with effect size = 8.39 and the minimal size of each set *n* = 2 in the case of power = 0.80.

#### 2.2.2. Comparison of Monochorionic and Dichorionic Postnatal Discordance

The differences in glycine, β-alanine, GABA, and asparagine levels in monochorionic pairs were negative (−17%, −25%, −43%, −24%), while in dichorionic pairs they were positive (+9.7%, +14%, +39%, +9.7%). For leucine, the difference in monochorionic pairs was +7%, whereas in dichorionic pairs it was −22% (FDR = 0.10). For tryptophan, the difference in monochorionic pairs was +29%, versus −0.18% in dichorionic pairs (*p* = 0.049) (Figure 4, Table 9). Point statistical significance was discriminated according to Hotelling’s test, with *p*-value = 0.004, effect size d = 3.35 and minimum size of each sample set *n* = 3 in the case of power = 0.80. The OPLS-DA model yielded R^2^X = 0.63, R^2^Y = 0.97, Q^2^Y = 0.51, *p*(R^2^Y) = 0.02, and *p*(Q^2^Y) = 0.11 according to permutation tests.

#### 2.2.3. Comparison of Postnatally Discordant and Non-Discordant Pairs

The differences in AABA in the postnatally discordant group was −43%, versus +0.99% in the non-discordant group; FDR = 0.006. For tryptophan, the difference in discordant pairs was +15%, versus −1.4% in the non-discordant group (FDR = 0.13). Proline showed +22% versus +1.4% (FDR = 0.12). The differences for serine, valine, citrulline, leucine and histidine did not reach statistical significance (FDR = 0.08, FDR = 0.16, FDR = 0.05, FDR = 0.59 and FDR = 0.49) (Figure 5, Table 10). Points statistically significant were discriminated according to Hotelling’s test, with *p* = 0.004, effect size d = 48, minimum size of sample set *n* = 2 and power of test = 0.80. The OPLS-DA model yielded R^2^X = 0.24, R^2^Y = 0.82, Q^2^Y = 0.50, *p*(R^2^Y) = 0.006, and *p*(Q^2^Y) = 0.006 according to permutation tests.

### 2.3. Over-Representation Analysis

Over-representation analysis with lists of the detected amino acids set as the background did not obtain pathways with FDR < 0.05.

Using the default background metabolite list, we obtained the 19 most important pathways (Table S2, Figure 6, Figure 7, Figure 8 and Figure 9).

In monochorionic postnatal discordance, pathways of Na^+^-dependent neurotransmitter transporters and amino acid transport across the plasma membrane (Reactome), as well as protein synthesis and absorption pathways (KEGG), were the most enriched (Figure 6).

In dichorionic postnatal discordance, the NO_2_-dependent IL-12 pathway in NK cells, the nitric oxide signaling pathway (Reactome), VEGFR1 and SHP2 signaling (PID), and VEGF-hypoxia-angiogenesis (BioCarta) were enriched (Figure 7).

For the differences between postnatally discordant and non-discordant pairs, pathways related to central carbon metabolism (KEGG), tRNA charging, leukotriene biosynthesis, and the γ-glutamyl cycle were enriched (Figure 8).

For the differences between monochorionic and dichorionic postnatal discordance, the mTOR signaling pathway (KEGG) was enriched (Figure 9).

### 2.4. Correlation Analysis

Inter-twin differences in AABA, serine, and leucine were positively correlated with differences in birth weight, length, and head circumference (R ranging from 0.45 to 0.62, *p* < 0.05). The difference in tryptophan was negatively correlated with the difference in birth weight (R = −0.41, *p* = 0.04). Mean levels of glycine and arginine correlated with maternal BMI (R = 0.53 and R = 0.48, respectively). Differences in AABA and GABA correlated with gestational age at delivery (R = −0.58 and −0.51, respectively) and placental weight (ρ = 0.49 and 0.44, respectively). Maternal age correlated with differences in glycine (R = 0.43, *p* = 0.04), β-alanine (R = 0.43, *p* = 0.04), leucine (R = −0.42, *p* = 0.048), and phenylalanine (R = −0.59, *p* = 0.003). The difference in serine correlated with the difference in the volume of nipple feeding (R = −0.66, *p* = 0.001) (Figure 10). Inter-twin differences in AABA and tryptophan were signature of sFGR and have a medium association with gestational age at one time point. All associations have FDR > 0.05.

Therefore, we added partial correlation analysis and confirmed the strong association of sFRG with discordance of AABA: R = −0.56, *p* = 0.007. In that time, the association of sFGR and discordance of tryptophan lost significance after removing involvement of gestational age: R = 0.21, *p* = 0.35.

### 2.5. Dynamics of Amino Acid Differences over Time

In monochorionic postnatally discordant pairs, difference between the first (1–2 days) and second (post-conceptional age 37–40 weeks) time points was observed for β-alanine (*p* = 0.03, FDR = 0.18), proline (*p* = 0.03, FDR = 0.18), asparagine (*p* = 0.04, FDR = 0.18), glutamic acid (*p* = 0.03, FDR = 0.82) and tryptophan (*p* = 0.01, FDR = 0.18). Notably, the direction of the difference for asparagine and tryptophan reversed. In dichorionic postnatally discordant pairs, differences were observed only for AABA (*p* = 0.04, FDR = 0.49) (Figure 11; Table 11 and Table 12). The first time point was collected before full enteral feeding was established and therefore primarily reflects the intrauterine metabolic state. The attenuation of the inter-twin differences by the second time point indicates temporal changes in the metabolic profile after birth; however, the design does not allow us to distinguish resolution of an sFGR-related abnormality from the effects of gestational maturation, postnatal nutrition, and neonatal adaptation.

## 3. Discussion

The present study provides a comprehensive analysis of the DBS amino acid profile in twin pairs with sFGR, accounting for chorionicity and detailed clinical characterization. The novelty of this work lies in the identification of divergent changes in the concentrations of key amino acids (glycine, tryptophan, leucine) in monochorionic versus dichorionic postnatal discordance. These differences are compatible with, but do not prove, the hypothesis of distinct pathophysiological mechanisms: intraplacental redistribution through vascular anastomoses, which is characteristic of monochorionic twins [16], and placental insufficiency, which is typical of dichorionic twins [17]. However, definitive confirmation of these mechanisms requires direct investigations of placental transport and inter-fetal blood flow, which were not performed in the present study. Beyond static inter-twin differences, the dynamics of these differences are noteworthy: postnatal remodeling of metabolic differences with a reversal of the direction for asparagine and tryptophan was observed in monochorionic twins, which may reflect metabolic plasticity in the early postnatal period.

It is important to distinguish between postnatal birth-weight discordance, which defined the study groups, and prenatal EFW discordance, which is one of the Delphi diagnostic criteria for sFGR. All 11 postnatally discordant pairs were retrospectively confirmed to fulfil the Delphi criteria for sFGR (Supplementary Table S1). However, the magnitude of postnatal discordance does not directly reflect the severity of prenatal sFGR, and the two measures should not be used interchangeably. Similarly, sFGR, SGA, and growth discordance describe related but distinct concepts: sFGR is a prenatal diagnosis, SGA is a postnatal phenotype (birth weight < 10th percentile), and discordance describes inter-twin discrepancy.

An advantage of this study is the use of DBS, the collection of which is integrated into routine neonatal practice in Russia. This enables retrospective analysis of already available samples without additional invasive procedures. Furthermore, recent studies have demonstrated the potential of DBS metabolic parameters for predicting bronchopulmonary dysplasia in preterm infants [18], confirming the broad potential of DBS methodology in neonatology and justifying further development of this approach. However, several significant amino acids (AABA, GABA, serine, asparagine, tryptophan, leucine) are not included in the standard expanded screening panel, necessitating an extension of the analyte panel. At the same time, it should be noted that DBS sample quality critically affects accuracy, and strict adherence to preanalytical protocols is essential [19].

### 3.1. Clinical Characteristics and Maternal Factors

The observed data on earlier delivery and lower placental weight in postnatally discordant pairs are consistent with established patterns. The absence of differences in clinical parameters between monochorionic and dichorionic pairs points to a similar phenotype of postnatal discordance, despite possible differences in the underlying mechanisms. The older parental age in the dichorionic group is in agreement with the literature data on the role of advanced age in the development of placental insufficiency [20]; in the monochorionic group, the leading role is likely attributable to vascular anastomoses; however, direct comparisons require further investigation.

### 3.2. Interpretation of Amino Acid Differences

Direct measurements of placental transport, vascular anastomoses, and intertwin blood flow were not performed in this study. Therefore, all mechanistic explanations proposed below are speculative and intended to generate hypotheses for future functional studies.

In monochorionic postnatally discordant pairs, the presence of arterio-venous anastomoses may lead to shunting of blood from the smaller to the larger twin, accompanied by amino acid transfer (Table 6, Figure 1). The larger twin showed elevated concentrations of most amino acids, whereas the smaller twin showed reduced concentrations, with the exception of proline and tryptophan, which may be utilized in the liver of the larger fetus or through other mechanisms. Importantly, the larger twin in monochorionic sFGR should not be regarded as an unaffected internal control: it shares the same placental circulation and is itself exposed to hemodynamic and metabolic consequences of the unbalanced anastomotic flow, including volume overload, cardiac strain, and polycythemia. Therefore, the higher concentrations observed in the larger twin cannot be interpreted as representing a normal physiological state; rather, they reflect the shared placental pathology. This is consistent with the hypothesis put forward by Bajoria et al. [16].

In dichorionic postnatally discordant pairs, the absence of anastomoses implies that each fetus receives amino acids exclusively from its own placental territory, and the observed differences reflect differential placental transport activity (Table 7, Figure 2). The elevated glycine level in the smaller twin may represent a compensatory antioxidant response, since glycine is a precursor of glutathione, although this remains a hypothesis [17]. This is consistent with current evidence that reduced activity of placental transport systems A and L is associated with fetal growth restriction, while their increased activity is associated with excessive fetal growth [17].

Thus, the comparison of metabolic response in monochorionic and dichorionic postnatal discordance demonstrated potential divergence: in monochorionic discordance, redistribution of most amino acids from the smaller to the larger twin is observed, whereas in dichorionic discordance, the differences are associated with autonomous regulation of placental transport, in which glycine may accumulate in the smaller twin, presumably as a compensatory antioxidant mechanism. The opposite trend of potential changes for key amino acids (glycine, tryptophan, leucine) across chorionicity types (Table 9, Figure 4) may demonstrate the necessity of stratification by chorionicity in metabolomic studies of sFGR. However, definitive conclusions regarding the mechanisms require further experimental investigations.

Comparison of postnatally discordant and non-discordant pairs identified AABA, serine, citrulline, and tryptophan as important parts of metabolite profile difference according to OPLS-discrimination analysis (Figure 5, Table 10). These results are consistent with the systematic review by Conde-Agudelo et al. [13], in which phenylalanine metabolism in newborn dried blood spots was identified as one of the key pathways associated with FGR/SGA. Importantly, AABA (α-aminobutyric acid) is a metabolite closely linked to phenylalanine metabolism and the glutathione cycle. In that time, discordance of tryptophan lost statistical significance of association with sFGR after adjustment for association with gestational age of delivery; their association with discordance must be checked in the future studies.

### 3.3. Over-Representation Analysis and Possible Molecular Mechanisms

The following interpretations of the most important enriched pathways are therefore hypothesis-generating.

The pathway analysis performed identified differences in enriched pathways depending on chorionicity type (Figure 6 and Figure 7). In monochorionic postnatal discordance, pathways of Na^+^-dependent neurotransmitter transporters and amino acid transport across the plasma membrane (Reactome), as well as protein synthesis and absorption pathways (KEGG), were some of the potentially more important pathways (Figure 6). In dichorionic postnatal discordance, among the potential interesting pathways were the NO_2_-dependent IL-12 pathway in NK cells, the nitric oxide signaling pathway (Reactome), VEGFR1 and SHP2 signaling (PID), and VEGF-hypoxia-angiogenesis (BioCarta) (Figure 7). These differences in the enriched pathways are compatible with the hypothesis that different molecular mechanisms may be engaged depending on the type of placentation. For the differences between postnatally discordant and non-discordant pairs, pathways related to central carbon metabolism (in KEGG terminology, “Central carbon metabolism in cancer”) were important. These pathways are related to fundamental processes of glycolysis and energy metabolism characteristic of proliferating cells. This is broadly consistent with recent data from Huang et al. [14], who reported similar enrichment in amniotic fluid analysis, as well as with pathways of tRNA charging, leukotriene biosynthesis, and the γ-glutamyl cycle (Figure 8).

The identification of mTOR signaling as a potential differential pathway between monochorionic and dichorionic pairs is consistent with, but does not prove, the current understanding of the key role of mTOR in regulating placental amino acid transport in abnormal growth [17,21,22]. However, all these interpretations remain hypothetical and warrant verification through functional studies. Also, the list of potentially interesting pathways was obtained by using the default background set in ORA, which made the results potentially more optimistic than realistic.

### 3.4. Correlations with Clinical Outcomes

The significant delay in the initiation of nipple feeding and in achieving full enteral volume observed in the smaller twin in postnatally discordant pairs is consistent with the literature data on the increased risk of oral feeding difficulties in children with growth restriction [23,24]. The moderate to strong correlation between the serine difference and feeding delay (R = −0.66) may support consideration of serine as a potential marker of functional gastrointestinal maturity. This is in line with the findings of Huang et al. [14] and Huang et al. [15], in which serine in the first trimester correlated with neurocognitive development, suggesting a possible prognostic value; however, this requires confirmation in prospective studies, taking into account the fact that the FDR of the correlation is higher than 0.05 (threshold of statistical significance).

### 3.5. Dynamics and Plasticity

The temporal pattern observed in this study provides insight into the origin of the metabolic differences. The first DBS sample was collected on days 1–2 of life, before the establishment of full enteral feeding, and therefore primarily reflects the intrauterine metabolic state. In monochorionic postnatally discordant pairs, a non-significant attenuation of within-pair differences was observed by the second time point (post-conceptional age 37–40 weeks) for β-alanine, glutamic acid, proline, asparagine, and tryptophan, with a reversal of the direction of the difference for asparagine and tryptophan (Figure 11a). In dichorionic postnatally discordant pairs, non-significant attenuation was observed only for AABA (Figure 11b). These findings may demonstrate temporal changes in the inter-twin amino acid differences between the early neonatal period and post-conceptional age 37–40 weeks. However, the design does not allow us to determine whether this attenuation reflects resolution of an sFGR-related metabolic abnormality or whether it reflects the combined effects of gestational maturation, postnatal nutritional exposure, and neonatal adaptation; all changes have an FDR > 0.05, even in the case of non-adjusted *p* < 0.05.

Two aspects of the design reduce, but do not eliminate, the influence of prematurity and postnatal factors. First, the second sample was collected at a comparable post-conceptional age in preterm and term infants. Second, the primary comparisons were made within twin pairs, which share gestational age at birth, postnatal age at sampling, and neonatal care conditions. These features make it unlikely that the observed attenuation is explained solely by differences in postnatal age at sampling. However, we cannot exclude that the longer duration of postnatal nutritional exposure in the more preterm discordant group contributed to the second time-point findings, and the design does not allow us to separate this from resolution of sFGR-related changes.

These observations are broadly consistent with the findings of Liu et al. [25] , who showed that chorionicity is associated with the metabolic phenotype as early as birth. Differences in pathways, particularly GSH and tryptophan metabolism, could be hypothesized to contribute to why postnatal remodeling in monochorionic twins occurs differently. The elimination of placental anastomoses after birth may contribute to the attenuation of these differences, especially for tryptophan. The authors also note that chorionicity may influence metabolic “cross-talk” between twins and is associated with birth weight discordance [25].

The results of the present study regarding postnatal remodeling are broadly consistent with the conclusions of Yang et al. [26], who analyzed the metabolomic profile of hair from newborn monochorionic twins with sFGR. It was shown that metabolite profiles in hair (which accumulate in utero) differ depending on the severity of sFGR (Gratacós types) and are associated with the average fetal growth rate after 28 weeks but not with birth weight. This indirectly supports, but does not confirm, the notion that the metabolic imprint of intrauterine disturbances does not necessarily directly correlate with birth weight, as postnatal factors (including nutrition and changes in placental function after birth) may rapidly alter metabolic status. In the study by Yang et al., markers (2-aminobutyric acid, cysteine, alanine, tyrosine) with AUC > 0.9 for discriminating severe forms of sFGR were also identified, which is consistent with, but does not prove, the significance of AABA found in the present study [26]. These longitudinal changes should not be interpreted as proof that sFGR-related metabolic abnormalities resolve; they reflect temporal changes in amino acid profiles influenced by multiple postnatal factors.

The data on postnatal changes obtained in the present study are in line with studies highlighting the high metabolic plasticity in preterm infants [27] and demonstrate the postnatal plasticity of amino acid profiles in twins with sFGR. Whether this plasticity reflects resolution of sFGR-related changes or maturation-related effects cannot be determined from the present data. This opens possibilities for early nutritional intervention; however, definitive conclusions require controlled studies.

Taken together, the longitudinal data demonstrate temporal changes in the inter-twin amino acid differences, but they do not establish that these changes represent specific resolution of an sFGR-related metabolic abnormality. Distinguishing between these possibilities will require studies with standardized nutritional protocols and serial sampling at closer intervals.

### 3.6. Limitations and Future Perspectives

The main limitations of this study are the small sample size (particularly the subgroup of monochorionic non-discordant pairs, *n* = 3), the inability to completely exclude the influence of postnatal nutrition at the second time point, and the lack of stratification by sFGR severity (presence or absence of Doppler abnormalities, reverse diastolic flow). The study groups were defined by postnatal birth-weight discordance, which is not equivalent to prenatal EFW discordance. Although all discordant pairs were retrospectively confirmed as sFGR, the use of postnatal grouping may have introduced selection bias. Preanalytical factors (hematocrit, drying time) were not fully controlled; however, within-pair comparisons mitigate their impact. OPLS-DA models, which are based on such a small sample size set, can be overfitting, despite satisfactory results of statistical tests; we must remember that this is just a pilot study, and sets of selected markers have only exploratory character as a direction for future studies.

Nevertheless, the study possesses considerable strength owing to the within-pair design, which minimizes the influence of genetic and maternal factors and allows the identified metabolic differences to be explored in relation to placental mechanisms. However, it should be emphasized that in monochorionic pregnancies the larger twin is not an unaffected control, as it shares the same placental circulation and is itself exposed to hemodynamic and metabolic consequences of the unbalanced anastomotic flow. Therefore, the observed differences reflect the shared placental pathology rather than a comparison between a normal and an abnormal twin. We did not directly assess placental transport, placental vascular anatomy, or intertwin blood flow. Consequently, the mechanistic interpretations proposed in this manuscript remain hypothetical and require validation in studies with direct placental and hemodynamic measurements.

Despite the aforementioned limitations, the identified associations between the levels of individual amino acids (serine, AABA, glycine/tryptophan ratio) and clinical outcomes may provide a foundation for the development of stratified patient management approaches in future studies. The use of early DBS amino acid profiling (as early as day 2–3 of life) may inform future hypothesis-driven studies. For instance, the glycine/tryptophan ratio in the smaller twin could be explored as a candidate marker of the severity of placental insufficiency. Interpretation of this index should take chorionicity into account: in monochorionic postnatal discordance, decreased glycine and increased tryptophan may be associated with more profound metabolic disturbances, whereas in dichorionic postnatal discordance, elevated glycine may reflect compensatory processes. Given the divergent nature of changes across placentation types, such an approach could be hypothesis-generating for future nutritional studies. For example, in monochorionic discordance with pronounced metabolic shifts, earlier initiation of parenteral nutrition might be considered in future studies, whereas in dichorionic discordance a less aggressive approach may be appropriate. Furthermore, the rapid postnatal normalization of differences in monochorionic twins may point to the potential effectiveness of early nutritional correction. Notably, in the study by Huang et al. [15], serine was included in a first-trimester metabolite combination correlating with long-term neurocognitive development, suggesting possible prognostic significance beyond the neonatal period. However, the present observational data do not demonstrate that amino acid abnormalities cause feeding difficulties or that amino-acid-guided nutritional intervention would improve neonatal outcomes. All these assumptions require verification in prospective interventional studies. The difference in gestational age at birth between the discordant and non-discordant groups (32 vs. 34 weeks) remains a limitation, as preterm birth itself may influence amino acid metabolism. However, the first DBS sample was collected before full enteral feeding, and the within-pair design controls for gestational age at birth. The second time point may be influenced by the longer duration of postnatal nutrition in the more preterm discordant group, and this should be considered when interpreting the postnatal remodeling data. Also, adjusted *p*-values of metabolite discordance at the first and the second time points increased higher than the threshold of significance, and we must interpret the obtained results carefully.

## 4. Materials and Methods

### 4.1. Study Design and Inclusion Criteria

The study was conducted in accordance with the Declaration of Helsinki and was approved by the local ethics committee (protocol No. 4 2021/04). All enrolled twin pairs were born at a single perinatal center between 2021 and 2024. Inclusion criteria were twin pregnancy (monochorionic or dichorionic, confirmed by ultrasound), live birth, and availability of dried blood spot samples collected on days 1–2 of life. Exclusion criteria were severe congenital malformations, chromosomal abnormalities, and confirmed intrauterine infection. The diagnosis of selective fetal growth restriction (sFGR) was established in accordance with the criteria of the international Delphi consensus [7]. According to these recommendations, for monochorionic twins, the diagnosis of sFGR was made when at least two of four additional criteria were present: estimated fetal weight (EFW) of one twin < 10th percentile, abdominal circumference of one twin < 10th percentile, EFW discordance ≥ 25%, and umbilical artery pulsatility index of the smaller fetus > 95th percentile. For dichorionic twins, at least two of three additional criteria were required: EFW of one twin < 10th percentile, EFW discordance ≥ 25%, and umbilical artery pulsatility index of the smaller fetus > 95th percentile. Furthermore, regardless of chorionicity, the presence of a single solitary criterion—EFW of one twin < 3rd percentile—was also considered sufficient for the diagnosis of sFGR [7]. The individual prenatal EFW and AC percentiles, EFW discordance, UA-PI values, and the specific Delphi criteria fulfilled for each discordant pregnancy are presented in Supplementary Table S1. A total of 23 twin pairs were included in the study. Postnatal birth-weight discordance ≥ 25% was used to define discordant pairs; 11 pairs were discordant (6 monochorionic, 5 dichorionic), and 12 pairs were non-discordant (3 monochorionic, 9 dichorionic). All 11 discordant pairs were retrospectively confirmed to fulfil the Delphi criteria for sFGR (Supplementary Table S1). The smaller twin in each pair was defined as the twin with the lower birth weight percentile. Mothers and fathers provided written informed consent to participate in the study.

In this study, two distinct measures of inter-twin growth discrepancy were used. Prenatal EFW discordance was calculated from ultrasound-derived EFW at diagnosis and served as one of the Delphi diagnostic criteria for sFGR. Postnatal birth-weight discordance was calculated from actual birth weights and was used to define the study groups. These measures are related but not interchangeable. The individual prenatal characteristics and the Delphi criteria fulfilled for each discordant pair are presented in Supplementary Table S1.

SGA was defined as birth weight < 10th percentile for gestational age, and severe SGA as birth weight < 3rd percentile, using INTERGROWTH-21st Newborn Size standards with sex-specific curves. The smaller twin in each pair was defined as the twin with the lower birth weight percentile.

### 4.2. Collection and Preparation of Dried Blood Spot Samples

Capillary blood samples were collected on days 1–2 of life, before the establishment of full enteral feeding, and therefore primarily reflect the intrauterine metabolic state. The second sample was collected upon reaching a post-conceptional age of 37–40 weeks, ensuring comparable maturity between preterm and term infants at the time of sampling. This design allows the separation of prenatal metabolic signatures from postnatal changes. Blood was applied to standard Whatman 903 filter paper cards, dried at room temperature for 2 h, and stored at −20 °C until analysis. This method yields stable samples suitable for long-term storage and is widely accepted in neonatal screening.

### 4.3. Amino Acid Analysis

Sample preparation was performed according to the following procedure: from each DBS sample, a 3 mm diameter disc was punched out; 480 µL of chloroform-methanol mixture (2:1, *v*/*v*) was added at 4 °C; the sample was subjected to ultrasonic treatment for 10 min; 150 µL of water was added; the mixture was vortexed for 5 min; the resulting solution was centrifuged at 13,000× *g* for 5 min at room temperature; 200 µL of the upper aqueous-methanolic layer was transferred; the extract was dried under a nitrogen stream for 30 min at 60 °C; 200 µL of 3N hydrochloric acid in butanol was added. The mixture was vortexed for 3 min; centrifuged at 13,000× *g* for 15 s at room temperature; incubated at 60 °C for 15 min to carry out the derivatization reaction; centrifuged at 13,000× *g* for 15 s at room temperature; dried under a nitrogen stream for 30 min at 60 °C; reconstituted in 200 µL of acetonitrile/water (1:1, *v*/*v*); vortexed for 5 min; and centrifuged at 13,000× *g* for 15 s at room temperature for 10 min. Then, 120 µL of the resulting sample was transferred to a vial with an insert for further analysis.

Amino acid levels in dried blood spot extracts were determined by high-performance liquid chromatography with mass spectrometric detection (HPLC-MS). The study employed an Agilent 1260 II liquid chromatograph (Agilent, Santa Clara, CA, USA) coupled with an Agilent 6460 mass spectrometer (Agilent, Santa Clara, CA ,USA). Chromatographic separation was performed on an Agilent Zorbax Eclipse XDB-C18 column (100 × 2.1 mm, 1.8 µm, Agilent, Santa Clara, CA, USA). Mobile phase A was 10 mM ammonium acetate in water, and mobile phase B was 100% acetonitrile. The flow rate was 150 µL/min, and the injection volume was 3 µL. The following optimized gradient elution program was used: at 0 min, the proportion of mobile phase B was 5% and held for 0.1 min; by 0.2 min, the proportion of B was linearly increased to 25%; by 5 min, B reached 30%; by 15 min, B was increased to 55%; by 20 min, to 70%; by 20.5 min, to 95%; 95% B was held until 25 min; at 25.5 min, B was returned to the initial 5% and the column was equilibrated until 40 min. The optimized mass spectrometer settings were as follows: drying gas temperature 150 °C, drying gas flow rate 10 L/min, nebulizer gas pressure 2.76 bar, sheath gas temperature 400 °C, sheath gas flow rate 10 L/min, and capillary voltage 2000 V. Isotopically labeled amino acid analogs (Cambridge Isotope Laboratories, Tewksbury, MA, USA) were used as internal standards; calibration was performed using the internal standard method with construction of multicomponent calibration curves. Quality control included daily checks of sensitivity, linearity (coefficient of determination > 0.99), and reproducibility (coefficient of variation < 10% for intra-assay and inter-assay precision). Concentrations of 23 amino acids were assessed (alanine, arginine, asparagine, aspartic acid, valine, histidine, glycine, glutamine, glutamic acid, leucine, methionine, ornithine, proline, serine, tyrosine, threonine, tryptophan, phenylalanine, citrulline, α-aminobutyric acid (AABA), β-alanine, 3-aminoisobutyric acid and γ-aminobutyric acid (GABA)). All analyses were performed in triplicate, and values were averaged. Intra-assay and inter-assay variability did not exceed 10%. For reducing variation, concentrations of amino acids were normalized on the total sum of concentrations of the measured amino acids.

### 4.4. Clinical Data Collection

The following parameters were extracted from medical records and the perinatal registry: birth weight, length, and head circumference; birth weight percentile for gestational age; Apgar scores at 1 and 5 min; sex; duration of non-invasive respiratory support (hours); timing of transfer from the neonatal intensive care unit (NICU) to the neonatal pathology unit (NPU) and from the NPU to discharge; initiation of nipple feeding (days of life) and time to full nipple feeding; discharge weight; and number of antibiotic courses. Maternal parameters included age, height, pre-pregnancy weight, gestational weight gain, body mass index (BMI), gestational age at delivery, placental weight, concomitant diseases (gestational diabetes mellitus, preeclampsia, anemia, hypertension, hypothyroidism, and others), and feeding type at discharge.

Prenatal EFW discordance was calculated according to the following formula:

(1) $$∆\mathrm{EFW}=(\frac{{\mathrm{EFW}}_{\mathrm{larger}}\;-{\;\mathrm{EFW}}_{\mathrm{smaller}}}{{\mathrm{EFW}}_{\mathrm{larger}}}\times 100)\%,$$

where *EFW_larger_* is the EFW of the larger newborn and *EFW_smaller_* is the EFW of the smaller newborn.

Postnatal birth-weight discordance was calculated according to the following formula:

(2) $$∆m=(\frac{2\times (m_{1}\;-{\;m}_{2})}{m_{1}\;+\;m_{2}}\times 100)\%,$$

where m_1_ is the weight of the smaller newborn and m_2_ is the weight of the larger newborn.

For each twin pair, the within-pair difference in clinical parameters was calculated using the following formula:

(3) $$∆P=(\frac{2\;\times \;(P_{1}\;-\;P_{2})}{P_{1}\;+\;P_{2}}\times 100)\%,$$

where *P*_1_ is the parameter value in the smaller newborn and *P*_2_ is the value in the larger newborn.

### 4.5. Statistical Analysis

To identify amino acids discriminating between samples from the larger and smaller twins within discordant pairs, orthogonal projections to latent structures (OPLS), with calculation of variable importance in projection (VIP), were used [28]. OPLS-DA models used one predictive axis and two orthogonal axes and were validated using internal 7-times cross-validation with 500 cycles of permutation. Discrimination of the sample sets was determined by Hotelling’s test. Minimal needed sizes of the sets were calculated based on effect size, threshold of significance 0.05 and minimal power 0.8 [29]. Effect size was calculated by the following formula:

(4) $$∆\;=\sqrt{{\left(d_{1}-d_{2}\right)}^{T}\Sigma^{-1}(d_{1}-d_{2})},$$

where d_1_ and d_2_ are vectors of center of each group, and Σ is the covariance matrix [29].

Amino acids with VIP > 1 were selected as important metabolites. For these amino acids, in the case of comparing between pairs, comparisons of levels between groups were performed using one-way analysis of variance (ANOVA), and in the case of intra-pair comparison, comparisons of levels were performed by paired *t*-tests.

Differences in amino acid levels within each twin pair were compared between postnatally discordant and non-discordant pairs. The difference in levels was calculated as

(5) $$∆I=(\frac{2\;\times \;(I_{1}-I_{2})}{I_{1}+I_{2}}\times 100)\%,$$

where I_1_ is the amino acid normalized level in the smaller newborn and I_2_ is the normalized level in the larger newborn. Comparisons of amino acid level differences between twins were performed for all postnatally discordant pairs versus non-discordant pairs as well as monochorionic discordant pairs versus dichorionic discordant pairs. Also, all 23 *p*-values from each comparison were adjusted using the Benjamini–Hochberg correction.

Analogous comparisons were performed for clinical parameters. Quantitative clinical parameters were analyzed using Mann–Whitney–Wilcoxon tests for comparing maternal parameters with between-group discordance and paired *t*-tests or Wilcoxon signed-rank tests according to their distribution in the case of comparing twin parameters, while categorical variables were analyzed using Fisher’s exact test.

Spearman’s correlation analysis was performed between amino acid normalized level differences and neonatal clinical parameter differences, between mean amino acid normalized levels in the pair and maternal clinical parameters, and between amino acid level differences and maternal clinical parameters. Also, *p*-values from correlation analysis were adjusted by Benjamini–Hochberg correction. For amino acids, to determine which discordance had statistically significant differences (*p* < 0.05) between the sFGR group and group without sFGR and had statistically significant association with gestational age at delivery, we performed partial correlation analysis using the Spearman method for amino acid discordance, gestational age of delivery and presence of sFGR, coding sFGR as a binary variable (1 in the case of presence of sFGR and 0 in case of no sFGR) and gestational age as the third variable.

Amino acids with VIP > 1 that differed between twins in each comparison group were included in over-representation pathway analysis using the ConsensusPathDB tool (http://cpdb.molgen.mpg.de/, accessed on 10 October 2026) with databases including HumanCyc, Reactome, KEGG, PID, BioCarta, and SMPDB, using default background lists and background lists based on lists of detected targets [30]. Pathways with FDR < 0.05 were considered statistically significantly enriched. Importance of pathways were calculated by harmonic mean of the ratio size of the set targeted metabolites to the full size of the pathway and (−log_10_(FDR)).

To assess the temporal dynamics of the inter-twin metabolic differences, amino acid level differences were compared between the first (days 1–2 of life, before the establishment of full enteral feeding) and second (post-conceptional age 37–40 weeks) time points. These comparisons were performed separately for the 6 monochorionic postnatally discordant and 5 dichorionic postnatally discordant pairs using paired *t*-tests. The first time point was considered to primarily reflect the intrauterine metabolic state, whereas the second time point reflected postnatal remodeling. Statistical significance was set at *p* < 0.05. *p*-values were adjusted by Benjamini–Hochberg correction.

All calculations were carried out in the R environment (version 4.3.3) [31] using the ropls package (version 1.34.0) [32] and hotelling (version 1.0-8) [33]. Visualization was performed using the ggplot2 (version 3.5.2) [34], reshape2 (version 1.4.4) [35], forcats (version 1.0.0) [36], and pheatmap (version 1.0.13) [37] packages.

## 5. Conclusions

The present study suggested that, in twin pairs with postnatal birth-weight discordance, chorionicity is associated with divergent changes in amino acid levels. In monochorionic postnatally discordant twins, the larger twin exhibited higher levels of glycine, AABA, GABA, serine, asparagine, and citrulline, whereas proline and tryptophan were reduced. In dichorionic postnatally discordant twins, the larger twin showed elevated AABA, valine, leucine, arginine, and citrulline, while glycine was higher in the smaller twin. Pathway analysis showed enrichment of NO-dependent signaling pathways in dichorionic postnatal discordance and amino acid transport pathways in monochorionic postnatal discordance.

Within-pair differences in several amino acids showed significant temporal changes by a post-conceptional age of 37–40 weeks, including attenuation of the differences for β-alanine, GABA, proline, asparagine, and tryptophan in monochorionic pairs and for AABA in dichorionic pairs, with a reversal of the direction of the difference for asparagine and tryptophan in monochorionic pairs. However, these temporal changes should not be interpreted as proof of normalization of an sFGR-related metabolic abnormality, as gestational maturation, postnatal nutrition, and neonatal adaptation may also contribute.

Clinically, postnatal birth-weight discordance was associated with delayed establishment of enteral feeding in the smaller twin. All postnatally discordant pairs were retrospectively confirmed to fulfil the Delphi criteria for sFGR. Among the 11 smaller twins from these pairs, seven (64%) were SGA at birth and five (45%) had severe SGA (<third percentile), whereas none of the 12 smaller twins from non-discordant pairs were SGA.

The use of dried blood spots, collected as part of neonatal screening, may serve as a candidate tool for future research aimed at early identification of at-risk infants and hypothesis-driven nutritional studies. Further prospective multicenter studies are required to confirm the clinical significance of the identified associations and to develop practical recommendations. The mechanistic explanations proposed are hypothesis-generating and should not be regarded as established. The present observational data cannot establish causality or clinical utility.

## Figures and Tables

**Figure 1:**
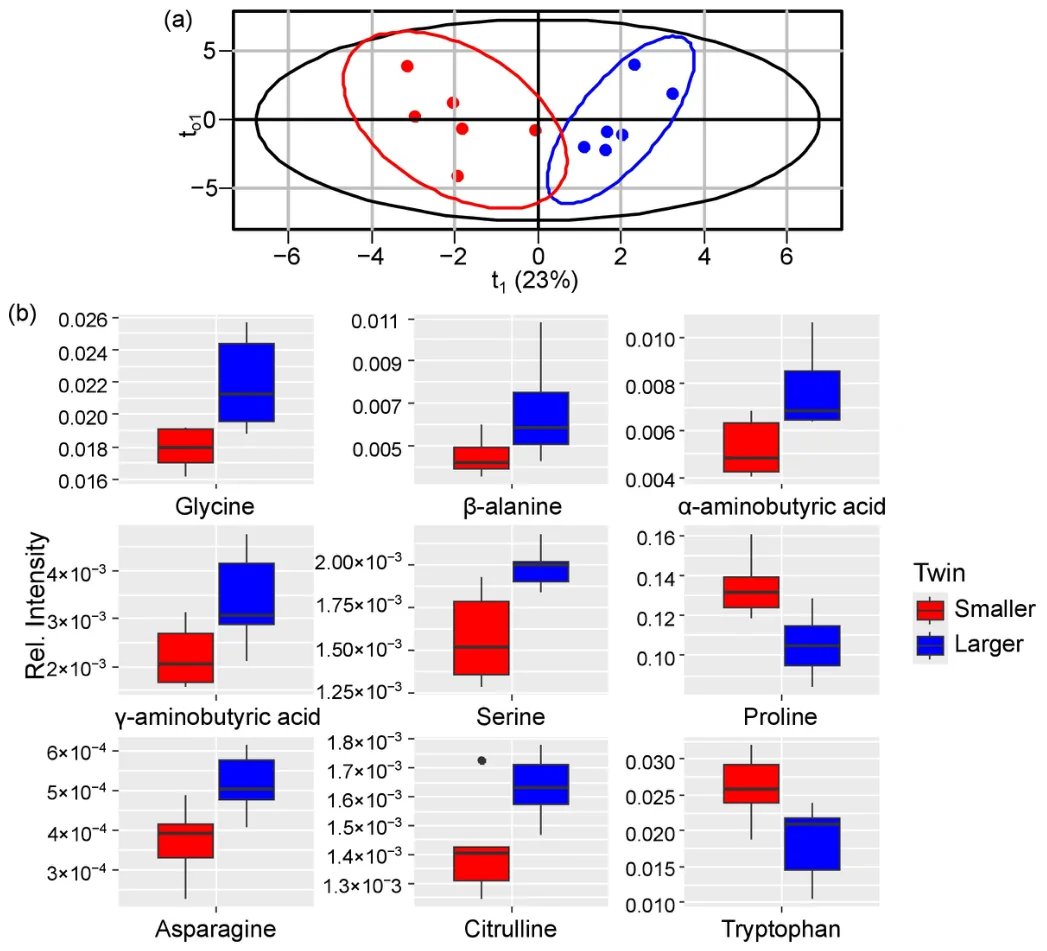
Comparison of amino acid profiles in DBS samples from newborns with higher (blue) and lower (red) birth weights in monochorionic postnatally discordant twin pairs: (**a**) score plot derived from OPLS analysis, ellipses are illustrated spaces (based on mean and sd values) of samples localization in the score plot: red is smaller twins, blue is larger twins, black is all samplles; (**b**) box plots showing the normalized levels of amino acids with VIP > 1. Outliers are labeled by black points.

**Figure 2:**
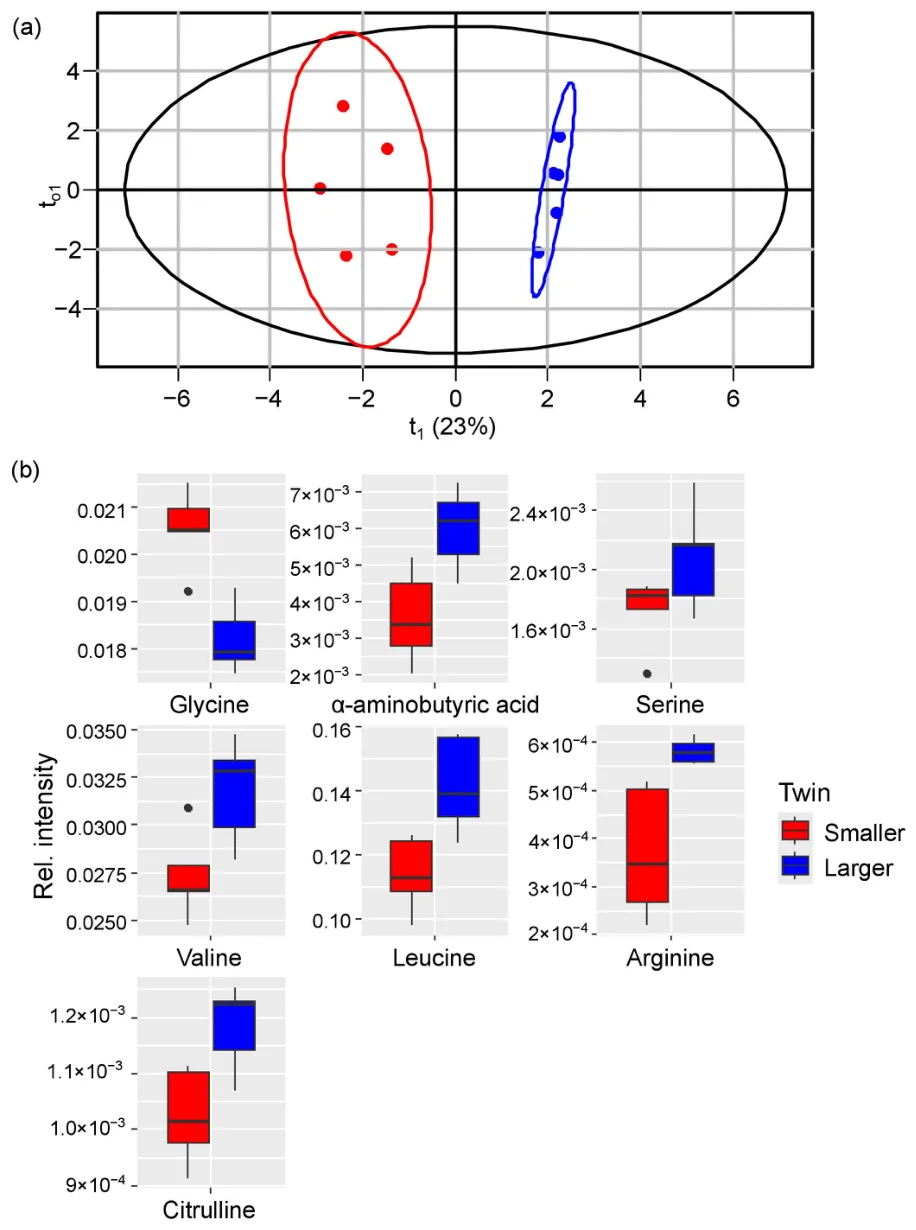
Comparison of amino acid profiles in DBS samples from newborns with higher (blue) and lower (red) birth weights in dichorionic postnatally discordant twin pairs: (**a**) score plot derived from OPLS analysis, ellipses are illustrated spaces (based on mean and sd values) of samples localization in the score plot: red is smaller twins, blue is larger twins, black is all samples; (**b**) box plots showing the normalized levels of amino acids with VIP > 1. Outliers are labeled by black points.

**Figure 3:**
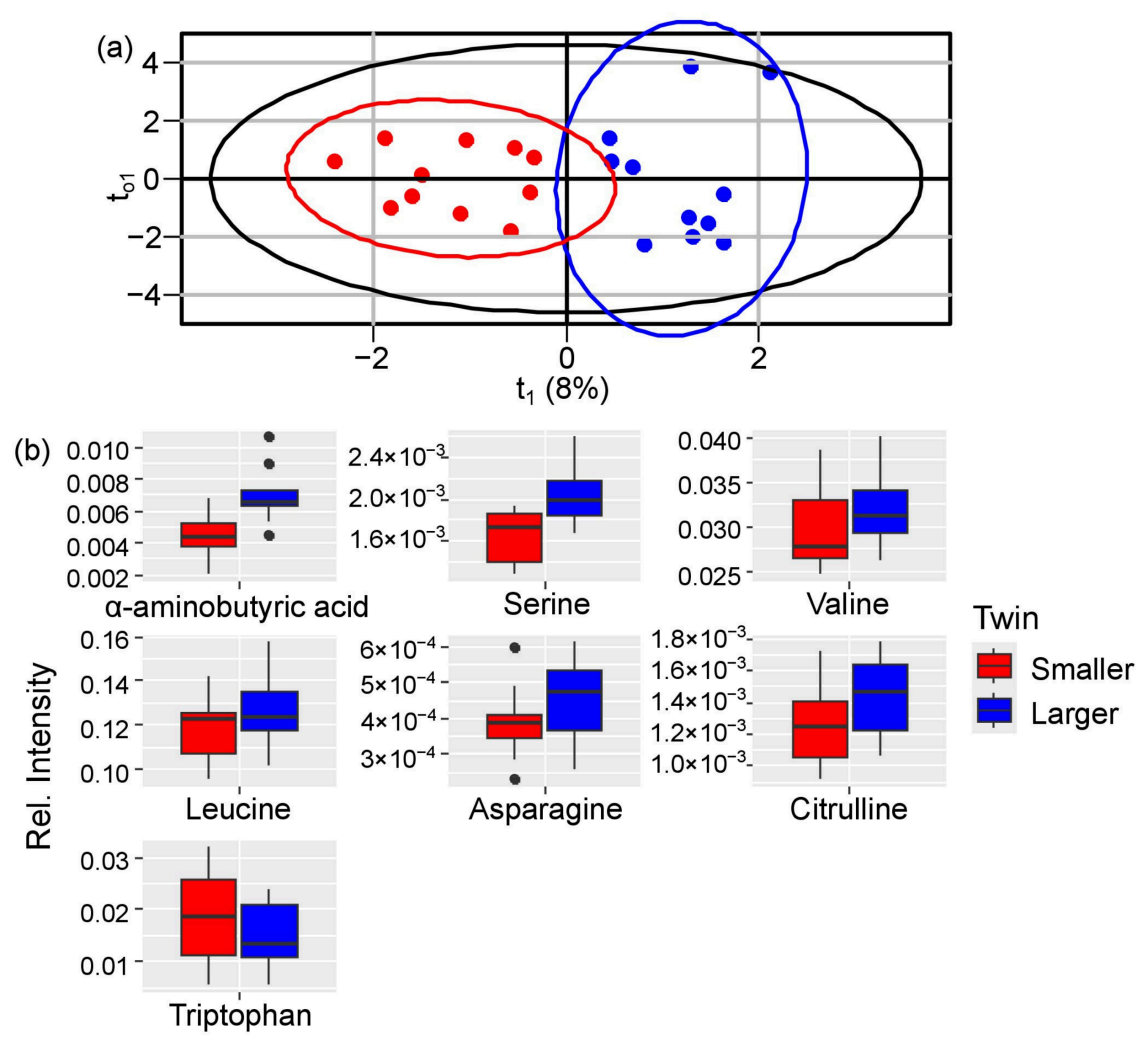
Comparison of amino acid profiles in DBS samples from newborns with higher (blue) and lower (red) birth weights in postnatally discordant twin pairs: (**a**) score plot derived from OPLS analysis, ellipses are illustrated spaces (based on mean and sd values) of samples localization in the score plot: red is smaller twins, blue is larger twins, black is all samples; (**b**) box plots showing the normalized levels of amino acids with VIP > 1. Outliers are labeled by black points.

**Figure 4:**
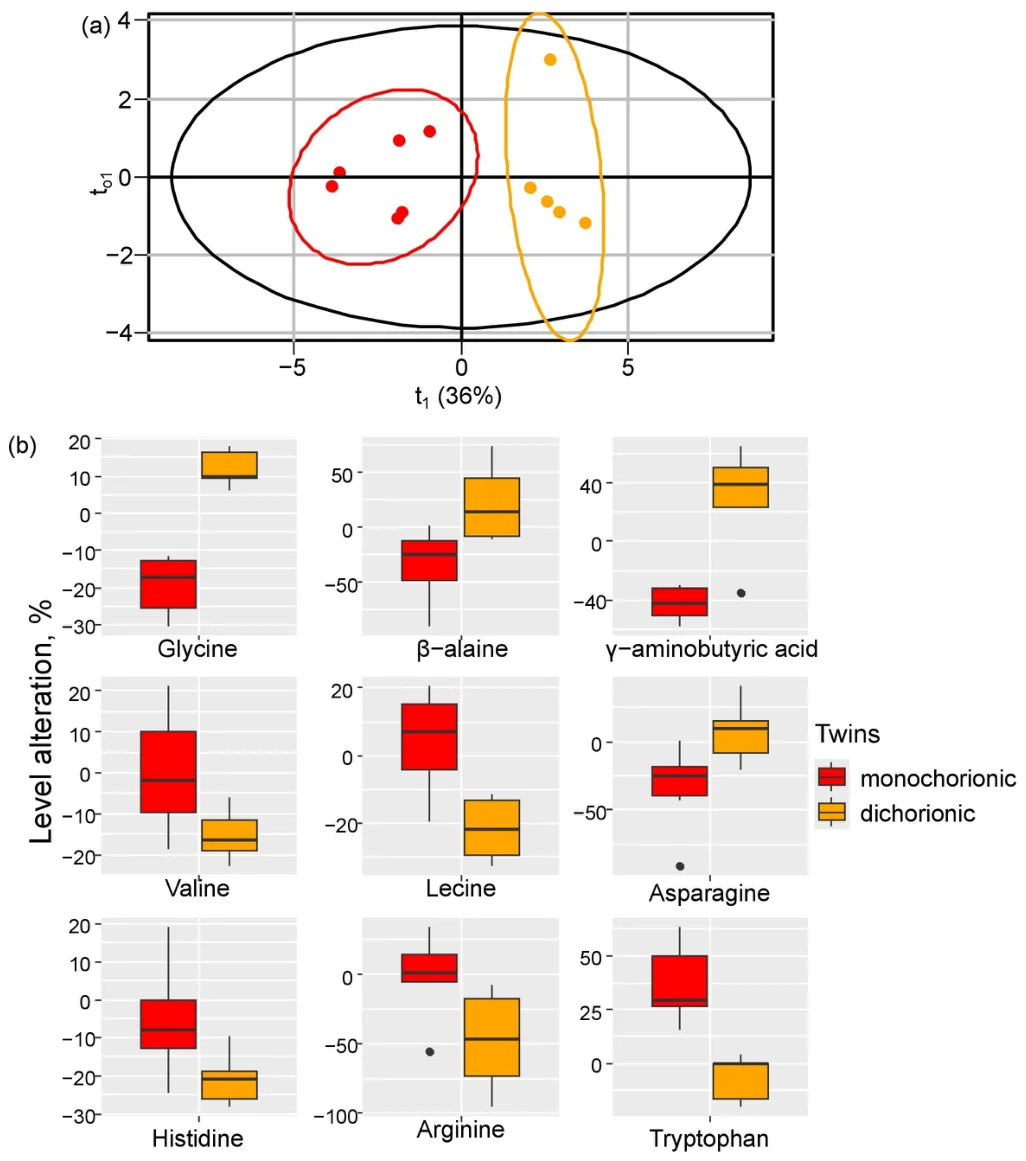
Comparison of amino acid profiles in DBS samples from newborns from postnatally discordant monochorionic (red) and dichorionic (orange) twin pairs: (**a**) score plot derived from OPLS analysis, ellipses are illustrated spaces (based on mean and sd values) of discordance localization in the score plot: red is discordance from monohorionic pairs, orange is discordance from monohorionic pairs, black is discordance from all pairs; (**b**) box plots showing the discordance of levels of amino acids $(\frac{2\;\times \;(I_{2}\;-\;I_{1})}{I_{1}\;+\;I_{2}}\times 100)\%$ with VIP > 1.

**Figure 5:**
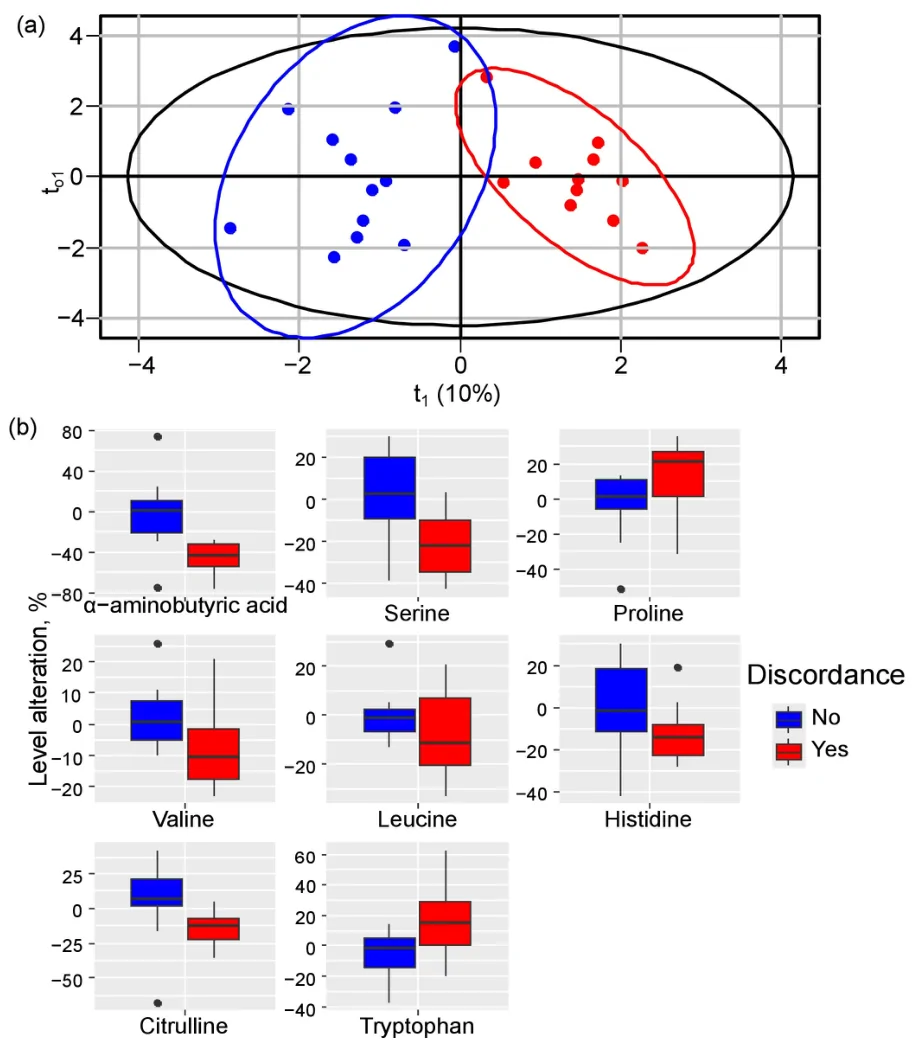
Comparison of amino acid profiles in DBS samples from newborns from twin pairs with postnatal birth-weight discordance less than 25% (blue) and greater than 25% (red): (**a**) score plot derived from OPLS analysis, , ellipses are illustrated spaces (based on mean and sd values) of dicordance localization in the score plot: red is pairs with discordance > 25%, blue is pairs with discordance < 25%, black is all samples; (**b**) box plots showing the discordance levels of amino acids between twins $(\frac{2\;\times \;(I_{2}\;-\;I_{1})}{I_{1}\;+\;I_{2}}\times 100)\%$ with VIP > 1. Outliiers are labeled by black point.

**Figure 6:**
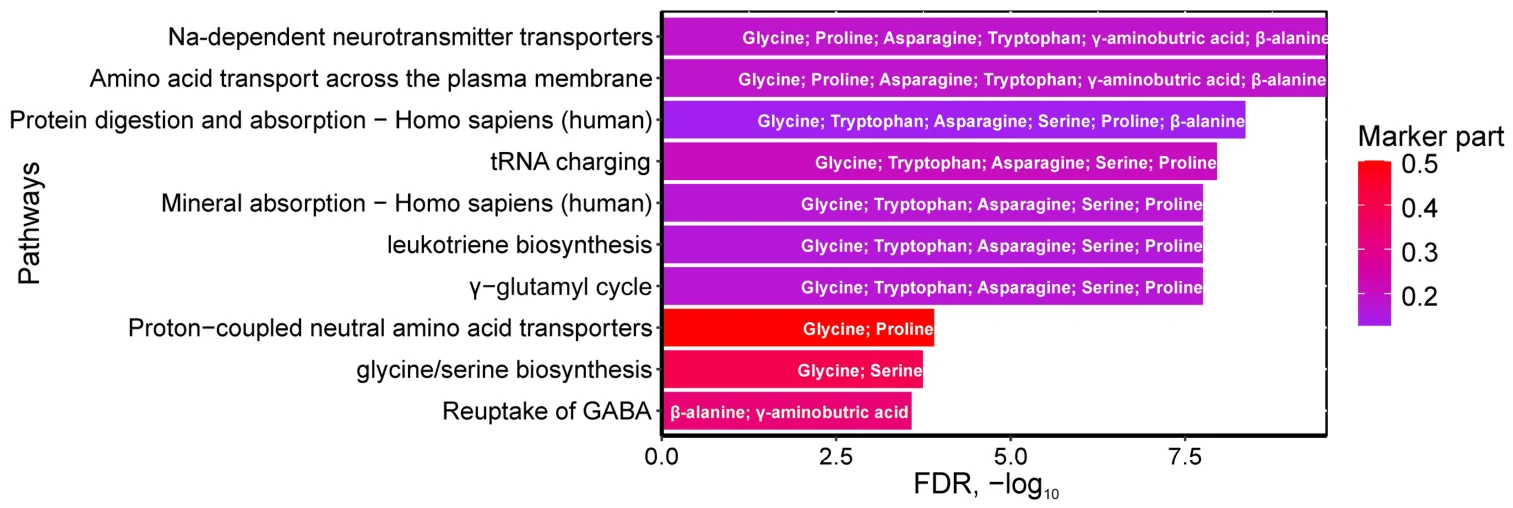
The ten most important pathways according to the harmonic mean of the ratio of the size of the set targeted metabolites to the full size of the pathway and (−log_10_(FDR)), which are enriched by amino acids that differed between infants in monochorionic postnatally discordant twin pairs based on default background set. Each pathway bar is labeled by the aminoacid, included in ORA-analysis and take part in pathways.

**Figure 7:**
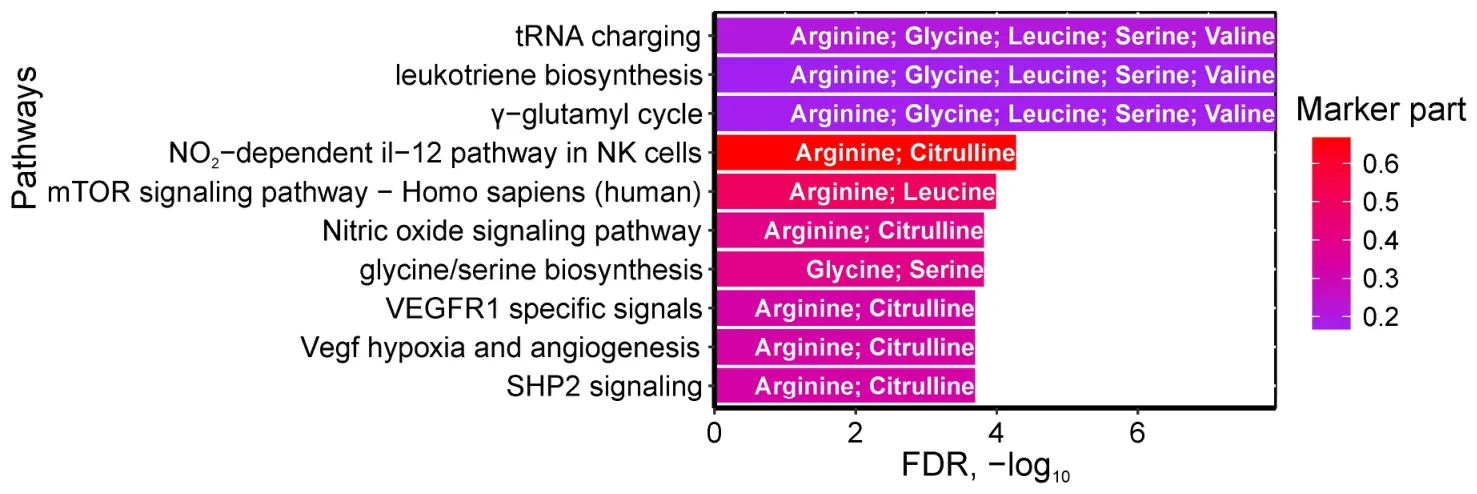
The ten most import pathways according to the harmonic mean of the ratio of the size of the set targeted metabolites to the full size of the pathway and (−log_10_(FDR)), which are enriched by amino acids that differed between infants in dichorionic postnatally discordant twin pairs based on default background set. Each pathway bar is labeled by the aminoacid, included in ORA-analysis and take part in pathways.

**Figure 8:**
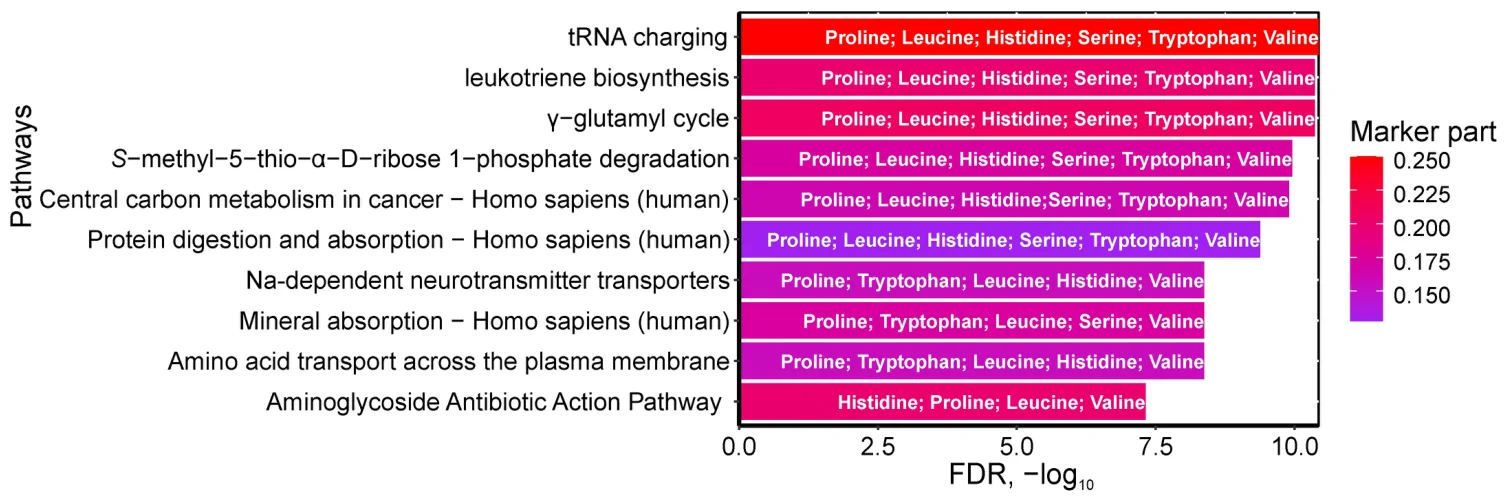
The ten most important pathways according to the harmonic mean of the ratio of the size of the set targeted metabolites to the full size of the pathway and (−log_10_(FDR)), which are enriched by amino acids whose inter-twin differences characterize the differences between postnatally discordant and non-discordant twin pairs based on default background set. Each pathway bar is labeled by the aminoacid, included in ORA-analysis and take part in pathways.

**Figure 9:**
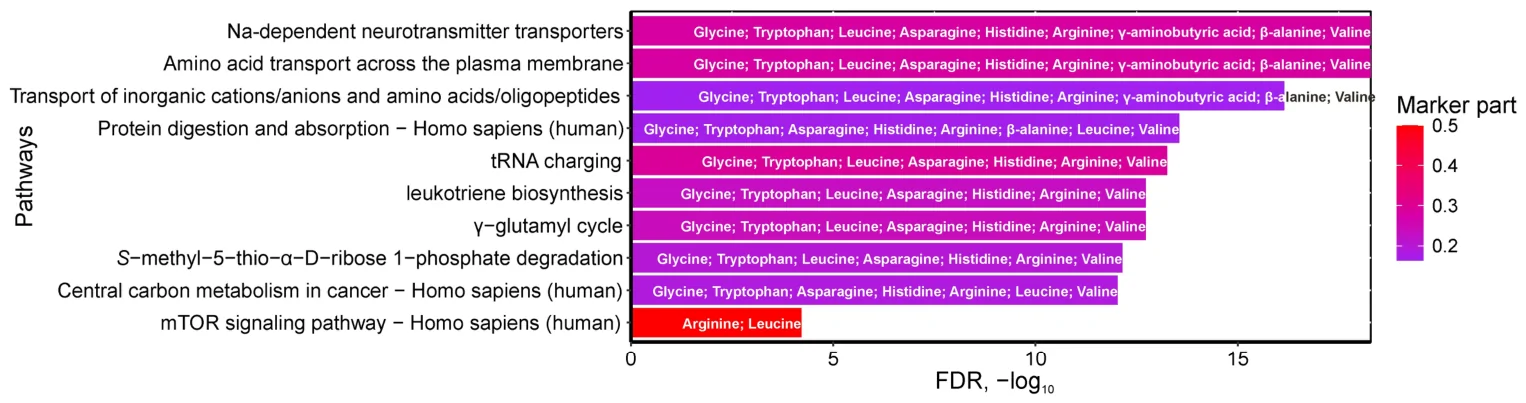
The ten most important pathways according to the harmonic mean of the ratio of the size of the set targeted metabolites to the full size of the pathway and (−log_10_(FDR)), which are enriched by amino acids, whose inter-twin differences characterize the differences between dichorionic postnatally discordant twin pairs and monochorionic postnatally discordant twin pairs based on default background set. Each pathway bar is labeled by the aminoacid, included in ORA-analysis and take part in pathways.

**Figure 10:**
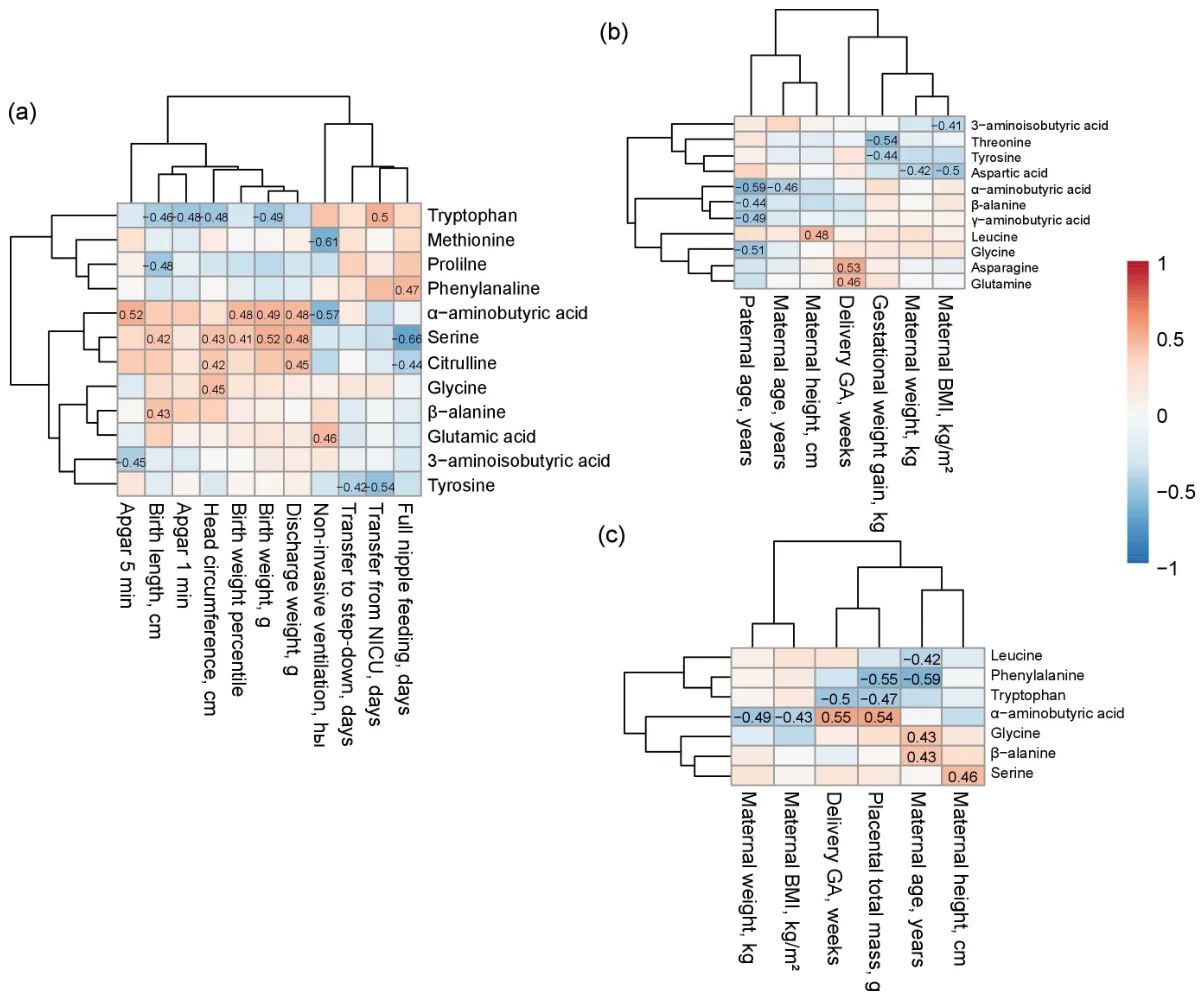
(**a**) Correlation plot of amino acid differences and differences in neonatal parameters in twin pairs. (**b**) Correlation plot of maternal clinical parameters and mean amino acid levels. (**c**) Correlation plot of maternal clinical parameters and inter-twin amino acid differences.

**Figure 11:**
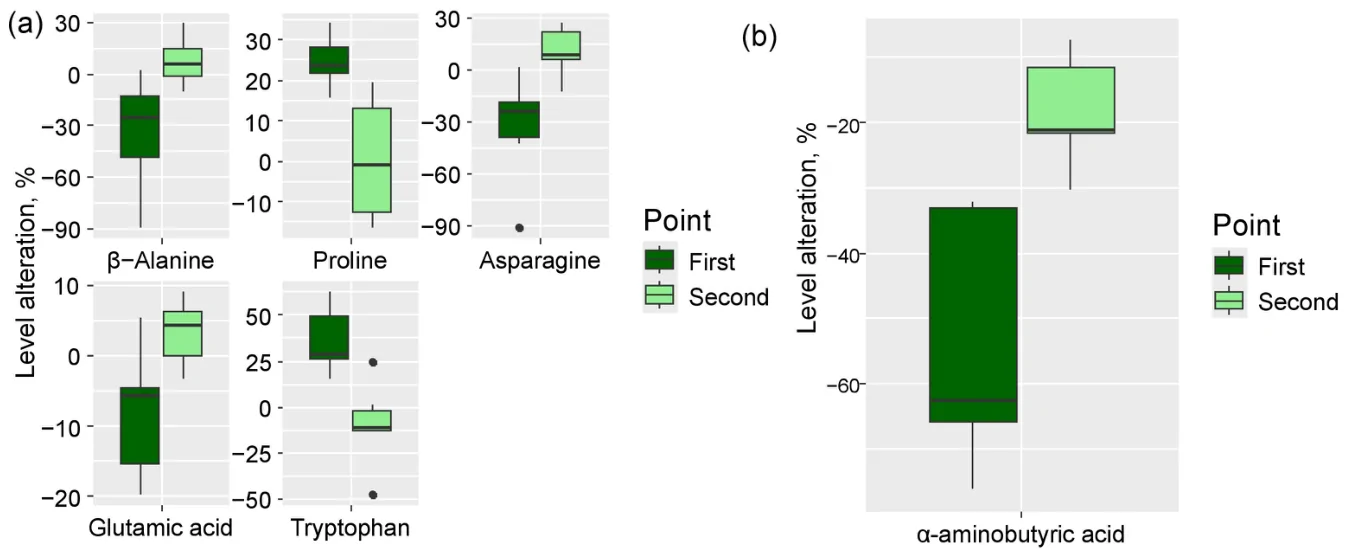
Discordance in amino acid levels $\left(\frac{2\times \;\left(I_{2}\;-\;I_{1}\right)}{I_{1}\;+\;I_{2}}\;\times 100\right)\%$ between (**a**) monochorionic discordant twins with non-adjusted *p* < 0.05 in between-point comparison; (**b**) dichorionic discordant twins with non-adjusted *p* < 0.05 in between-point comparison. Outliers are labeled by black points.

**Table 1:** Clinical parameters of twins in postnatally discordant pairs (all retrospectively confirmed as sFGR). Parameters with statistically significant differences are shown in bold.

Parameter	Smaller Co-Twin (*n* = 11)	Larger Co-Twin (*n* = 11)	*p*-Value
**Birth weight, g**	**1160 (988; 1449)**	**1840 (1606; 2132)**	**<0.001**
**Birth length, cm**	**38 (36; 40)**	**42 (41; 44)**	**<0.001**
Non-invasive respiratory support, h	64 (40; 94)	68 (34; 88)	0.68
Age at NICU discharge, days of life	6 (4.5; 8.5)	5 (4.5; 9.5)	0.20
Age at transfer to general neonatal ward, days of life	6 (4.5; 8.5)	5 (4.5; 9.5)	0.28
Initiation of nipple feeding, days of life	18 (16; 21)	12 (10; 18)	**0.04**
Time to full nipple feeding, days of life	32 (20; 38)	21 (14; 34)	**0.007**
**Discharge weight, g**	**1935 (1801; 1980)**	**2510 (2449; 2727)**	**<0.001**
**Head circumference at birth, cm**	**28 (27; 30)**	**31 (29; 32)**	**0.004**
**Birth weight percentile**	**5 (1; 10)**	**60 (45; 72)**	**<0.001**
Apgar score at 1 min	7 (6; 7)	7 (7; 7)	**0.02**
Apgar score at 5 min	8 (7; 8)	8 (7; 8)	0.13
Male sex, n (%)	2 (18%)	3 (27%)	0.68
Number of antibiotic courses, n (%)	0–4 (36%)	0–2 (18%)	0.20
	1–4 (36%)	1–6 (55%)	
	2–1 (9%)	2–1 (9%)	
	3–1 (9%)	3–2 (18%)	
	4–1 (9%)	4–0 (0%)	

**Table 2:** Differences in clinical parameters between twins in postnatally discordant and non-discordant twin pairs. Statistically significant differences are shown in bold.

Parameter	Non-Discordant (*n* = 12)	Discordant Pairs (*n* = 11)	*p*-Value
**Postnatal birth-weight discordance, %**	**−9.4 (−15; −4.8)**	**−40 (−48; −28)**	**<0.001**
**Birth length difference, %**	**−2.3 (−4.3; −2.1)**	**−10 (−15; −6.2)**	**<0.001**
Non-invasive respiratory support difference, %	−2.3 (−22; 3.1)	−2 (−10; 29)	0.68
Age at NICU discharge difference, %	0 (−64; 0)	6.7 (0; 18)	0.20
Age at transfer to general ward difference, %	0 (−14; 0)	0 (0; 18)	0.28
**Initiation of nipple feeding difference, %**	**0 (−14; 0)**	**30 (15; 42)**	**0.04**
**Time to full nipple feeding difference, %**	**0 (−7; 0)**	**22 (7.9; 30)**	**0.007**
**Discharge weight difference, %**	**−9.8 (−12; −4.5)**	**−26 (−35; −22)**	**<0.001**
**Head circumference at birth difference, %**	**−3 (−3.1; 0)**	**−7.1 (−10; −3.6)**	**0.004**
**Birth weight percentile difference, %**	**−52 (−98; −20)**	**−156 (−191; −143)**	**<0.001**
Apgar score at 1 min difference, %	0 (0; 14)	0 (−15; 0)	**0.02**
Apgar score at 5 min difference, %	0 (0; 12)	0 (−6.7; 0)	0.13

**Table 3:** Differences in clinical parameters between twins in postnatally discordant monochorionic and dichorionic twin pairs.

Parameter	Monochorionic Discordant (*n* = 6)	Dichorionic Discordant (*n* = 5)	*p*-Value
Postnatal birth-weight discordance, %	−42 (−51; −40)	−28 (−37; −27)	0.17
Birth length difference, %	−13 (−17; −9.7)	−7.1 (−10; −5.4)	0.25
Non-invasive respiratory support difference, %	−2 (−8.1; 27)	−1.9 (−11; 35)	0.91
Age at NICU discharge difference, %	6.7 (0; 17)	9.1 (−12; 26)	1.00
Age at transfer to general ward difference, %	6.7 (0; 17)	0 (0; 18)	1.00
Initiation of nipple feeding difference, %	24 (4; 42)	32 (25; 40)	0.76
Time to full nipple feeding difference, %	22 (21; 30)	11 (3.1; 27)	0.48
Discharge weight difference, %	−24 (−38; −22)	−26 (−29; −23)	0.79
Head circumference at birth difference, %	−8.8 (−13; −4.4)	−6.7 (−9; −3.8)	0.46
Birth weight percentile difference, %	−183 (−192; −160)	−153 (−156; −133)	0.54
Apgar score at 1 min difference, %	0 (−12; 0)	0 (−15; 0)	0.84
Apgar score at 5 min difference, %	0 (0; 10)	0 (−13; 0)	0.19

**Table 4:** Parental clinical parameters associated with pregnancy course in non-discordant and postnatally discordant twin pairs. Statistically significant differences are shown in bold.

Parameter	Non-Discordant Pairs (*n* = 12)	Discordant Pairs (*n* = 11)	*p*-Value
Maternal age, years	36 (31; 39)	34 (30; 38)	0.63
Maternal height, cm	164 (163; 173)	169 (162; 171)	0.88
Maternal weight, kg	71 (67; 75)	85 (71; 89)	0.75
Gestational weight gain, kg	11 (11; 13)	11 (9; 20)	0.72
Maternal BMI, kg/m^2^	26 (24; 28)	29 (26; 31)	0.58
**Gestational age at delivery, weeks**	**34 (33; 36)**	**32 (32; 33)**	**0.01**
**Total placental weight, g**	**625 (575; 769)**	**544 (506; 598)**	**0.02**
Paternal age, years	39 (32; 43)	37 (35; 38)	0.65
GDM, n (%)	1 (8%)	5 (45%)	0.12
Intrahepatic cholestasis of pregnancy, n (%)	1 (8%)	1 (9%)	1.00
Cytolysis syndrome, n (%)	0 (0%)	1 (9%)	0.47
OHSS, n (%)	0 (0%)	1 (9%)	0.47
Anemia, n (%)	2 (17%)	2 (18%)	1.00
Hypertension, n (%)	1 (8%)	2 (18%)	0.59
Hypothyroidism, n (%)	1 (8%)	0 (0%)	1.00
Obesity, n (%)	0 (0%)	1 (9%)	0.96
Colpitis, n (%)	1 (8%)	0 (0%)	0.47
Chronic pyelonephritis, n (%)	0 (0%)	0 (0%)	1.00
Preeclampsia, n (%)	3 (25%)	2 (18%)	1.00
Number of chorions, n (%)	1–3 (25%)	1–6 (55%)	0.09
	2–9 (75%)	2–5 (45%)	
Number of amniotic sacs, n (%)	1–0 (0%)	1–1 (9%)	0.21
	2–12 (100%)	2–10 (91%)	
Feeding type at discharge, n (%)	Mixed—10 (83%)	Mixed—8 (73%)	0.96
	Artificial—0 (0%)	Artificial—1 (9%)	
	Breastfeeding—2 (17%)	Breastfeeding—2 (18%)	

**Table 5:** Parental clinical parameters associated with pregnancy course in monochorionic and dichorionic postnatally discordant pregnancies. Statistically significant differences are shown in bold.

Parameter	Monochorionic Discordant (*n* = 6)	Dichorionic Discordant (*n* = 5)	*p*-Value
**Maternal age, years**	**30 (27; 34)**	**39 (36; 40)**	**0.01**
Maternal height, cm	166 (162; 170)	172 (168; 172)	0.38
Maternal weight, kg	79 (71; 90)	86 (67; 86)	0.81
Gestational weight gain, kg	11 (8.8; 18)	13 (10; 18)	0.81
Maternal BMI, kg/m^2^	29 (27; 31)	28 (23; 30)	0.46
Gestational age at delivery, weeks	32 (32; 32)	33 (32; 34)	0.79
Total placental weight, g	506 (466; 556)	586 (544; 611)	0.19
**Paternal age, years**	**35 (32; 37)**	**40 (37; 41)**	**0.02**
GDM, n (%)	2 (33%)	3 (60%)	0.57
Intrahepatic cholestasis of pregnancy, n (%)	0 (0%)	1 (20%)	0.45
Cytolysis syndrome, n (%)	1 (17%)	0 (0%)	1.00
OHSS, n (%)	0 (0%)	1 (20%)	0.45
Anemia, n (%)	0 (0%)	2 (40%)	0.35
Hypertension, n (%)	1 (17%)	1 (20%)	1.00
Obesity, n (%)	1 (17%)	0 (0%)	1.00
Preeclampsia, n (%)	1 (17%)	1 (20%)	1.00
Feeding type at discharge, n (%)	Mixed—4 (67%)	Mixed—4 (80%)	1.00
	Artificial—1 (17%)	Artificial—0 (0%)	
	Breastfeeding—1 (17%)	Breastfeeding—1 (20%)	

**Table 6:** Amino acid normalized levels, presented as “Median” (“the 1st quartile”; “the 3rd quartile”), contributing the most to the separation of samples from the smaller and larger twins in monochorionic postnatally discordant pairs; fold change (FC), *p*-value (paired *t*-test), and variable importance in projection (VIP) derived from OPLS analysis.

Amino Acid	Smaller Twin	Larger Twin	FC	*p*	FDR	VIP
Glycine	1.8 × 10^−2^ (1.7 × 10^−2^; 1.9 × 10^−2^)	2.1 × 10^−2^ (2.0 × 10^−2^; 2.4 × 10^−2^)	1.18	0.004	0.02	1.60
β-Alanine	4.2 × 10^−3^ (4.0 × 10^−3^; 4.9 × 10^−3^)	5.9 × 10^−3^ (5.1 × 10^−3^; 7.5 × 10^−3^)	1.39	0.09	0.22	1.21
α-Aminobutyric acid (AABA)	4.8 × 10^−3^ (4.2 × 10^−3^; 6.3 × 10^−3^)	6.9 × 10^−3^ (6.5 × 10^−3^; 8.6 × 10^−3^)	1.43	<0.001	0.006	1.49
γ-Aminobutyric acid (GABA)	2.0 × 10^−3^ (1.7 × 10^−3^; 2.7 × 10^−3^)	3.1 × 10^−3^ (2.9 × 10^−3^; 4.2 × 10^−3^)	1.51	0.001	0.01	1.28
Serine	1.5 × 10^−3^ (1.4 × 10^−3^; 1.8 × 10^−3^)	2.0 × 10^−3^ (1.9 × 10^−3^; 2.0 × 10^−3^)	1.31	0.01	0.048	1.73
Proline	0.13 (0.12; 0.14)	0.1 (0.095; 0.11)	0.80	<0.001	0.001	1.38
Asparagine	3.9 × 10^−4^ (3.3 × 10^−4^; 4.1 × 10^−4^)	5.1 × 10^−4^ (4.8 × 10^−4^; 5.8 × 10^−4^)	1.29	0.048	0.16	1.64
Citrulline	1.4 × 10^−3^ (1.3 × 10^−4^; 1.4 × 10^−4^)	1.6 × 10^−3^ (1.6 × 10^−3^; 1.7 × 10^−3^)	1.16	0.07	0.20	1.70
Tryptophan	2.6 × 10^−2^ (2.4 × 10^−2^; 2.9 × 10^−2^)	2.1 × 10^−2^ (1.4 × 10^−2^; 2.2 × 10^−2^)	0.81	<0.001	0.007	1.21

**Table 7:** Amino acid normalized levels, presented as “Median” (“the 1st quartile”; “the 3rd quartile”), contributing the most to the separation of samples from the smaller and larger twins in dichorionic postnatally discordant pairs; fold change (FC), *p*-value (paired *t*-test), and variable importance in projection (VIP) derived from OPLS analysis.

Amino Acid	Smaller Twin	Larger Twin	FC	*p*	FDR	VIP
Glycine	2.1 × 10^−2^ (2.0 × 10^−2^; 2.1 × 10^−2^)	1.8 × 10^−2^ (1.8 × 10^−2^; 1.9 × 10^−2^)	0.87	0.006	0.04	1.89
α-Aminobutyric acid (AABA)	3.4 × 10^−3^ (2.8 × 10^−3^; 4.5 × 10^−3^)	6.2 × 10^−3^ (5.3 × 10^−3^; 6.7 × 10^−3^)	1.84	<0.001	0.01	1.46
Serine	1.8 × 10^−3^ (1.7 × 10^−3^; 1.9 × 10^−3^)	2.2 × 10^−3^ (1.8 × 10^−3^; 2.2 × 10^−3^)	1.19	0.06	0.17	1.27
Valine	2.7 × 10^−2^ (2.6 × 10^−2^; 2.8 × 10^−2^)	3.3 × 10^−2^ (3.0 × 10^−2^; 3.3 × 10^−2^)	1.24	0.006	0.04	1.39
Leucine	0.11 (0.11; 0.12)	0.14 (0.13; 0.16)	1.23	0.008	0.04	1.60
Arginine	3.5 × 10^−4^ (2.7 × 10^−4^; 5.0 × 10^−4^)	5.8 × 10^−4^ (5.6 × 10^−4^; 6.0 × 10^−4^)	1.67	0.03	0.11	1.71
Citrulline	1.0 × 10^−3^ (9.8 × 10^−4^; 1.1 × 10^−4^)	1.2 × 10^−3^ (1.1 × 10^−3^; 1.2 × 10^−3^)	1.20	0.01	0.04	1.70

**Table 8:** Amino acid normalized levels, presented as “Median” (“the 1st quartile”; “the 3rd quartile”), contributing the most to the separation of samples from the smaller and larger twins in all postnatally discordant pairs; fold change (FC), *p*-value (paired *t*-test), and variable importance in projection (VIP) derived from OPLS analysis.

Amino Acid	Smaller Twin	Larger Twin	FC	*p*	FDR	VIP
α-Aminobutyric acid (AABA)	4.3 × 10^−3^ (3.7 × 10^−3^; 5.2 × 10^−3^)	6.5 × 10^−3^ (6.3 × 10^−3^; 7.2 × 10^−3^)	1.51	<0.001	<0.001	2.33
Serine	1.7 × 10^−3^ (1.4 × 10^−3^; 1.9 × 10^−3^)	2.0 × 10^−3^ (1.8 × 10^−3^; 2.2 × 10^−3^)	1.15	<0.001	0.009	2.45
Valine	2.8 × 10^−2^ (2.7 × 10^−2^; 3.3 × 10^−2^)	3.1 × 10^−2^ (2.9 × 10^−2^; 3.4 × 10^−2^)	1.12	0.15	0.50	1.01
Leucine	0.12 (0.11; 0.13)	0.12 (0.12; 0.14)	1.01	0.19	0.50	1.39
Asparagine	3.9 × 10^−4^ (3.4 × 10^−4^; 4.1 × 10^−4^)	4.7 × 10^−4^ (3.7 × 10^−4^; 5.4 × 10^−4^)	1.23	0.14	0.50	1.29
Citrulline	1.2 × 10^−3^ (1.1 × 10^−3^; 1.4 × 10^−3^)	1.5 × 10^−3^ (1.2 × 10^−3^; 1.6 × 10^−3^)	1.18	0.004	0.04	1.24
Tryptophan	1.9 × 10^−2^ (1.1 × 10^−2^; 2.6 × 10^−2^)	1.4 × 10^−2^ (1.1 × 10^−2^; 2.1 × 10^−2^)	0.73	0.03	0.19	1.11

**Table 9:** Discordance in amino acid levels $(\frac{2\;\times \;(I_{2}\;-\;I_{1})}{I_{1}\;+\;I_{2}}\times 100)\%$ contributing the most to the separation of monochorionic and dichorionic discordant twin pairs, *p*-value (Mann–Whitney test), and variable importance in projection (VIP), as derived from OPLS analysis.

Amino Acid	Discordance in MCDA, %	Discordance in DCDA, %	*p*	FDR	VIP
Glycine	−17 (−26; −13)	9.7 (9.6; 17)	0.004	0.049	1.61
β-Alanine	−25 (−48; −13)	14 (−7.8; 44)	0.03	0.10	1.25
γ-Aminobutyric acid (GABA)	−43 (−50; −32)	39 (23; 51)	0.01	0.10	1.36
Valine	−2 (−9.9; 10)	−16 (−19; −12)	0.08	0.16	1.02
Leucine	7 (−4.4; 15)	−22 (−30; −13)	0.02	0.10	1.42
Asparagine	−24 (−39; −18)	9.7 (−8; 15)	0.03	0.10	1.12
Histidine	−7.9 (−13; 0.015)	−21 (−26; −19)	0.05	0.69	1.20
Arginine	1.4 (−5; 14)	−47 (−73; −17)	0.03	0.10	1.20
Tryptophan	29 (26; 50)	−0.18 (−16; 0.14)	0.004	0.049	1.60

**Table 10:** Discordance in amino acid levels $(\frac{2\;\times \;(I_{2}\;-\;I_{1})}{I_{1}\;+\;I_{2}}\times 100)\%$ contributing the most to the separation of discordant and non-discordant twin pairs, *p*-value (Mann–Whitney test), and variable importance in projection (VIP) as derived from OPLS analysis.

Amino Acid	Discordance in Normal Pairs, %	Discordance in sFGR Pairs, %	*p*	FDR	VIP
α-Aminobutyric acid (AABA)	0.99 (−20; 10)	−43 (−54; −33)	<0.001	0.006	2.13
Serine	2.7 (−9.3; 20)	−22 (−34; −10)	0.01	0.08	1.81
Proline	1.4 (−6.1; 11)	22 (1.5; 27)	0.02	0.12	1.16
Valine	0.73 (−4.9; 7.3)	−11 (−18; −1.3)	0.04	0.16	1.07
Leucine	−0.86 (−6.7; 2.7)	−11 (−21; 7)	0.26	0.59	1.01
Histidine	−1.8 (−12; 18)	−14 (−23; −7.9)	0.15	0.49	1.07
Citrulline	7.2 (1.6; 21)	−12 (−22; −7.1)	0.004	0.05	1.79
Tryptophan	−1.4 (−14; 4.7)	15 (−0.02; 29)	0.03	0.13	1.67

**Table 11:** Discordance in amino acid levels $(\frac{2\times \;(I_{2}\;-\;I_{1})}{I_{1}\;+\;I_{2}}\;\times 100)\%$ between twins in discordant monochorionic pairs at the first and second time points.

Amino Acid	Discordance at 1st Point, %	Discordance at 2nd Point, %	*p*
Glycine	−17 (−26; −13)	−3.3 (−5; 1.1)	0.17
β-Alanine	−25 (−48; −13)	5.9 (−1; 14)	0.03
Alanine	−12 (−24; 2.7)	−5.1 (−11; 4.3)	0.76
α-Aminobutyric acid (AABA)	−41 (−45; −33)	−30 (−32; 16)	0.10
γ-Aminobutyric acid (GABA)	−43 (−50; −32)	−10 (−15; −7.4)	0.05
3-Aminoisobutyric acid	−3.1 (−17; 7.1)	−4.4 (−9.3; 17)	0.45
Serine	−28 (−35; −11)	−21 (−36; 17)	0.46
Proline	23 (22; 28)	−0.97 (−12; 13)	0.03
Valine	−2 (−9.9; 10)	−2.3 (−9.7; −1.8)	0.70
Threonine	−0.82 (−6; 1.4)	10 (−1.3; 25)	0.08
Leucine	7 (−4.4; 15)	−5.3 (−9.1; −1.3)	0.21
Asparagine	−24 (−39; −18)	8.1 (6; 22)	0.04
Ornithine	0.66 (−19; 9.9)	−9.2 (−26; 0.28)	0.61
Glutamine	0.4 (−18; 10)	3 (−4.3; 13)	0.74
Methionine	−13 (−33; 19)	0.85 (−11; 8.4)	0.90
Histidine	−7.9 (−13; 0.015)	3.5 (−4.9; 33)	0.62
Phenylalanine	6.4 (−0.031; 20)	−3.1 (−8.7; 5.8)	0.16
Arginine	1.4 (−5; 14)	−22 (−71; 17)	0.33
Citrulline	−11 (−26; −6.3)	−25 (−46; 12)	0.91
Tyrosine	0.14 (−1.5; 16)	−2.2 (−8.4; 23)	0.65
Aspartic acid	−1.2 (−10; 15)	2.7 (−19; 14)	0.98
Glutamic acid	−5.6 (−15; −4.6)	4.4 (−0.036; 6.2)	0.03
Tryptophan	29 (26; 50)	−11 (−13; −1.8)	0.01

**Table 12:** Discordance in amino acid levels $(\frac{2\times \;(I_{2}\;-\;I_{1})}{I_{1}\;+\;I_{2}}\;\times 100)\%$ between twins in discordant dichorionic pairs at the first and second time points. *p*-values are shown for the comparison between time points.

Amino Acid	Discordance at 1st Point, %	Discordance at 2nd Point, %	*p*	FDR
Glycine	9.7 (9.6; 17)	5.4 (4.1; 7.7)	0.30	0.49
β-Alanine	14 (−7.8; 44)	0.5 (−19; 17)	0.36	0.49
Alanine	−4.5 (−5.1; 25)	−9 (−9.5; 0.015)	0.26	0.49
α-Aminobutyric acid (AABA)	−63 (−66; −33)	−21 (−22; −11)	0.04	0.49
γ-Aminobutyric acid (GABA)	39 (23; 51)	−4.6 (−30; 4.8)	0.32	0.49
3-Aminoisobutyric acid	36 (27; 46)	21 (2.1; 42)	0.69	0.72
Serine	−16 (−34; −14)	−17 (−23; −16)	0.64	0.70
Proline	−3.3 (−3.4; 6.2)	7.3 (−0.43; 9.3)	0.80	0.80
Valine	−16 (−19; −12)	−7.3 (−9.9; −7.1)	0.11	0.49
Threonine	7 (−2.4; 23)	7.2 (−0.53; 7.8)	0.19	0.49
Leucine	−22 (−30; −13)	−9.4 (−16; −1.2)	0.27	0.49
Asparagine	9.7 (−8; 15)	−16 (−20; 1.3)	0.21	0.49
Ornithine	−0.24 (−1.8; 2.4)	0.038 (−7.7; 4.3)	0.28	0.49
Glutamine	12 (0.91; 12)	−1.2 (−1.3; 7.3)	0.35	0.49
Methionine	−15 (−42; −3.2)	14 (7.5; 15)	0.06	0.49
Histidine	−21 (−26; −19)	−11 (−15; −2.2)	0.16	0.49
Phenylalanine	−14 (−20; −3.6)	6.2 (−14; 8.7)	0.16	0.49
Arginine	−47 (−73; −17)	−36 (−44; −33)	0.55	0.63
Citrulline	−12 (−22; −11)	12 (−30; 16)	0.31	0.49
Tyrosine	−14 (−18; −13)	14 (−5.4; 23)	0.15	0.49
Aspartic acid	−7.6 (−44; −5.3)	−2.6 (−7.5; −0.66)	0.51	0.62
Glutamic acid	5.9 (0.37; 15)	−1.8 (−2.5; 6.4)	0.36	0.49
Tryptophan	−0.18 (−16; 0.14)	7.6 (−13; 16)	0.47	0.60

## Data Availability

The original contributions presented in this study are included in the article. Further inquiries can be directed at the corresponding author.

## Supplementary Materials

The following supporting information can be downloaded at: https://www.mdpi.com/article/10.3390/ijms27209156/s1.

## Abbreviations

The following abbreviations are used in this manuscript:

AABA	α-aminobutyric acid
ANOVA	Analysis of variance
BMI	Body mass index
DCDA	Dichorionic diamniotic
DBS	Dried blood spot
EFW	Estimated fetal weight
GABA	γ-aminobutyric acid
FDR	False discovery rate
HPLC-MS	High-performance liquid chromatography
MCDA	Monochorionic diamniotic
NICU	Neonatal intensive care unit
NPU	Neonatal pathology unit
OPLS-DA	Orthogonal projections to latent structures discriminant analysis
(s)FGR	(selective)Fetal growth restriction
SGA	Small for gestational age
VIP	Variable importance in projection
